# How to improve data quality in dog eye tracking

**DOI:** 10.3758/s13428-022-01788-6

**Published:** 2022-06-09

**Authors:** Soon Young Park, Kenneth Holmqvist, Diederick C. Niehorster, Ludwig Huber, Zsófia Virányi

**Affiliations:** 1 Comparative Cognition, Messerli Research Institute, University of Veterinary Medicine Vienna, Vienna, Austria; 2 Medical University Vienna, Vienna, Austria; 3 University of Vienna, Vienna, Austria; 4 Institute of Psychology, Nicolaus Copernicus University in Torun, Torun, Poland; 5 Department of Psychology, Regensburg University, Regensburg, Germany; 6 Department of Computer Science and Informatics, University of the Free State, Bloemfontein, South Africa; 7 Lund University Humanities Lab and Department of Psychology, Lund University, Lund, Sweden

**Keywords:** Eye tracking, Dogs, Eye movement, Data quality

## Abstract

Pupil–corneal reflection (P–CR) eye tracking has gained a prominent role in studying dog visual cognition, despite methodological challenges that often lead to lower-quality data than when recording from humans. In the current study, we investigated if and how the morphology of dogs might interfere with tracking of P–CR systems, and to what extent such interference, possibly in combination with dog-unique eye-movement characteristics, may undermine data quality and affect eye-movement classification when processed through algorithms. For this aim, we have conducted an eye-tracking experiment with dogs and humans, and investigated incidences of tracking interference, compared how they blinked, and examined how differential quality of dog and human data affected the detection and classification of eye-movement events. Our results show that the morphology of dogs’ face and eye can interfere with tracking methods of the systems, and dogs blink less often but their blinks are longer. Importantly, the lower quality of dog data lead to larger differences in how two different event detection algorithms classified fixations, indicating that the results of key dependent variables are more susceptible to choice of algorithm in dog than human data. Further, two measures of the Nyström & Holmqvist (*Behavior Research Methods, 42*(4), 188–204, 2010) algorithm showed that dog fixations are less stable and dog data have more trials with extreme levels of noise. Our findings call for analyses better adjusted to the characteristics of dog eye-tracking data, and our recommendations help future dog eye-tracking studies acquire quality data to enable robust comparisons of visual cognition between dogs and humans.

## Introduction

In the last decade, pupil (P) and corneal reflection (CR)-based eye-tracking systems (P–CR eye-tracking systems) that were initially developed to track human eyes (Merchant, 1967) have become a popular tool to study visual cognition in dogs [*Canis lupus familiaris*] (Somppi et al.,, 2012, 2014, 2016, 2017; Törnqvist et al.,, 2015, 2020; Téglás et al.,, 2012; Kis et al.,, 2017; Gergely et al.,, 2019; Barber et al., 2016; Correia-Caeiro et al.,, 2020, 2021; Ogura et al.,, 2020; Völter et al.,, 2020). The non-histological morphology of dog pupils and corneas is similar to that of humans, i.e., dogs have a circular dark pupil and a transparent reflective cornea (Malmström & Kröger, 2006; Banks et al., 2015; Nautscher et al., 2016). This similarity enables researchers to record the eye movements of dogs non-invasively using a remotely placed infrared camera. Using the same P–CR eye-tracking systems, and similar experimental designs and stimuli to those of human eye-tracking studies, the above studies have set the ground for cross-species comparisons of visual cognition between humans and dogs. However, the studies have also revealed important methodological challenges in dog eye tracking, which make such cross-species comparisons less straightforward. The most highlighted challenge across the studies is collecting dog eye-tracking data (raw data, the data before event classification through algorithms) of a quality that is comparable to that of human adult data.

The quality of eye-tracking data is often assessed in terms of the following three crucial properties of a segment of eye-movement data: accuracy, precision, and the amount of data loss (e.g., Holmqvist et al.,, 2012; Niehorster et al.,, 2020a, 2020a; Holmqvist, 2015; Holmqvist & Andersson 2017). First, data may be inaccurate, i.e., there is an offset between the real gaze location and the gaze position estimated by the eye-tracking system. Reported accuracy is typically determined by means of calibration and validation procedures performed on the experiment’s participants. Second, data may exhibit poor spatial precision, i.e., there is variation in the estimated gaze position despite the actual gaze position of the recorded eye being kept constant. Finally, data may exhibit a significant amount of invalid or lost data, for instance when the eye tracker cannot reliably detect and track the pupil or the corneal reflection center. Among the three properties, issues of data loss and inaccuracy stand out in dog eye tracking. Most previous dog eye-tracking studies reported “unreadable” or “insufficient” data for analysis or lack of data within trials (Somppi et al.,, 2012, 2014, 2016, 2017; Barber et al.,, 2016; Correia-Caeiro et al.,, 2020, 2021; Gergely et al.,, 2019; Kis et al.,, 2017; Téglás et al.,, 2012). Previous dog eye-tracking studies reported excluding on average about 15% (and some even over 50%, see Table 1) of data due to issues with data loss or other data-quality issues, which is higher than that commonly seen in eye-tracking studies with human adults (on average 9%, ranging from 3% to 15%, Holmqvist & Andersson, 2017, p.167). Park et al., (2020) reported that dog data has, despite calibration training, also poorer accuracy than that of humans, e.g., the average offset in the estimated gaze position measured during calibration and validation procedures was larger in dogs (0.88^∘^) than in humans (0.51^∘^).

**Table 1:** Overview of dog eye-tracking studies noting methodological details relevant for data quality

Study	Training	Eye-Tracking	Eye-video	Eye-Tracking	Recording duration	Attrition rate (%)	Trials excluded
		system	availability	data output	(per stimulus)	(= subjects excluded	/ Trial total×100
				used		/subject total	
Somppi et al., (2012)	chinrest	SMI RED 250			2 s	none reported	≈ 7*%* of total frames
Somppi et al., (2014)				fixation	1.5 s	≈ 6*%*	≈ 13*%* of total images
Somppi et al., (2016)			available		1.5 s	≈ 6*%*	≈ 7*%* of total images
Somppi et al., (2017)			(not default)		7 s	≈ 7*%*	≈ 2*%* of total images
Törnqvist et al., (2015)				fixation, saccade	2.5 s	≈ 13*%*	≈ 7*%* of total images
Törnqvist et al., (2020)					3 s	none reported	≈ 8*%* of total images
Téglás et al., (2012)	none	Tobii X50	unavailable	gaze	not applicable	≈ 53*%*	≈ 68*%* of total trials (condition average)
Kis et al., (2017)				gaze	5 s	≈ 47*%*	none reported
Gergely et al., (2019)				gaze (referred to as look)	12 s	≈ 36*%*	none reported
Barber et al., (2016)	chinrest,		available	fixation	5 s	none reported	≈ 37*%* of total trials
Park et al., (2020)	calibration,	EyeLink 1000	(default)	saccade, fixation	7 s	≈ 39*%*	≈ 49*%* (of total
	validation						saccades and fixations)
Völter et al., (2020)				gaze	16 s (exp. 1)	≈ 23*%*	none reported
					24 s (exp. 2)		
Ogura et al., (2020)	chinrest,	ETL-300-HD,	available	fixation	1.5 s	≈ 57*%*	none reported
	calibration	ISCAN	(default)				
Correia-Caeiro et al., (2020)	none	EyeLink 1000 Plus	available	fixation	5-12 s	≈ 4*%*	≈ 3*%* of total trials
Correia-Caeiro et al., (2021)		with head tracking	(default)		6.30 s (5–8 s)	≈ 8*%*	1-5 missing trials in 61 participants

More details of eye video are in the Method section. Gaze (5th column) refer to the data output without classification of eye-movement events through an algorithm

### Reasons for data loss and inaccuracy

#### Head movements and calibration difficulty

As to why the unusable or missing data occurred, some of the dog eye-tracking studies suggested “technical problems”, “software problems”, or “dog’s behavior (head movement)” (Somppi et al.,, 2012, 2014, 2016, 2017; Törnqvist et al.,, 2015, 2020). Other studies have described it as the loss of dogs’ “attention” or “eye-tracker signal” (Correia-Caeiro et al.,, 2020, 2021). We have also observed that most dogs tend to make more small head movements than human adults who have received clear verbal instruction to minimize their head movement. Thus, dog eye-tracking data overall suffer more from head movement-induced tracking loss or noise. A further factor reducing accuracy is that it is difficult to carry out the calibration and validation procedures with dogs, since they are not able to follow verbal instructions as human adults. Several dog eye-tracking studies reported exclusion of subjects due to calibration difficulty which increased their attrition rate (Téglás et al.,, 2012; Kis et al.,, 2017; Ogura et al.,, 2020; Somppi et al., 2014, 2016, 2017; Park et al.,, 2020) (Table 1). Eye-tracking studies that recorded from human infants (Hessels and Hooge, 2019) and non-human primates (Hopper et al., 2020) share similar challenges with dog eye tracking, as the subjects have behavioral tendencies and communication limits analogous to those of dogs. Hessels et al., (2015) and Niehorster et al., (2018), and Holmqvist et al., (2021) experimentally tested the effects of large and small head movements of either infants or adults on data quality in a selection of eye-tracking systems, and found that head movements caused systematic data loss due to tracking failure as well as increased inaccuracy and imprecision of the recorded data.

While there have not been counterpart studies in dogs, based on the shared challenges, comparable data quality can be expected between dogs and infants. Infants do not have the morphological characteristics of dogs that can interfere with tracking of the systems, while many dogs can on the other hand be trained to stay still on the chinrest to prevent data loss and inaccurate tracking due to head movement. Currently available data do not allow comparing data quality between infants and dogs, and each participant group thus comes with its unique challenges that careful experimental design may or may not be able to mitigate.

There have also been various efforts in dog eye tracking to prevent data loss and inaccurate tracking due to head movement. For instance, many studies have trained dog subjects for staying still on a chinrest (Somppi et al.,, 2012, 2014, 2016, 2017; Törnqvist et al.,, 2015; Barber et al.,, 2016; Park et al.,, 2020; Ogura et al.,, 2020; Völter et al.,, 2020; Törnqvist et al.,, 2020) (Table 1). Further, Barber et al., (2016) and Park et al., (2020), and Völter et al., (2020) pre-experimentally trained dogs also for the calibration and validation procedures, while Somppi et al., (2012, 2014, 2016, 2017) and Törnqvist et al., (2015, 2020) ran the calibration procedure and the experimental task on separate days (using the SMI RED 250 eye-tracking system) to reduce the risk that dogs lose interest and display a lack of vigilance during the experimental task. While special attention has been paid to the behavior and training of dogs, it has not been explored whether and how factors other than unwanted movements and a lack of vigilance could further lead to tracking loss and inaccuracy in dog data.

#### Dog unique morphology

In human eye-tracking practice, it is well known that certain vision-related or ophthalmic morphology of subjects affects the ability of P–CR eye-tracking systems to track participants and the accuracy of the data, and therefore, pre-selection of subjects based on such morphological characteristics is advisable. For example, Hessels et al., (2015) reported that accuracy and precision are worse, and more data loss occurs for infants with bluish than dark iris color. Likewise, Holmqvist (2015) suggests that, statistically speaking, it is best to recruit tall, male participants in the age around 17–25 years, who have big, brown eyes with pupils that are not too large, forward- or upward-pointing eye lashes, and dark hair (see also Nyström et al.,, 2013), Despite the overall similarity in the eye morphology between humans and dogs, some morphological characteristics of dogs differ from those of humans and non-human primates (summarized in Table 2), and may thus pose unique challenges for P–CR systems. The eye-feature detection and tracking mechanisms of current eye-tracking systems are built predominantly for the eyes of humans and non-human primates, and cannot be assumed to work equally well when tracking dog eyes. Indeed, indications of tracking interference due to morphological characteristics can be found in some of the previous dog eye-tracking studies. For example, (Kis et al., 2017) excluded some dogs due to not only their inattentiveness but also their head shape (too long nose, lateral position of the eyes), which made it impossible to carry out the calibration procedure for both eyes at the same time. Park et al., (2020) pre-selected dogs based on some of the morphological characteristics described in Table 2 to reduce tracking loss. Yet, so far no studies have systematically investigated if and how morphological characteristics unique to dogs could interfere with the tracking performance of the P–CR eye trackers. Should the characteristics interfere with tracking of dog eyes, sharing the experience and relevant information among dog eye-tracking researchers would be helpful for future studies (see also Hopper et al., (2020) for a similar endeavor with non-human primates).

### Factors affecting eye-movement event classification

In most eye-tracking studies, the analysis of the collected eye-movement data starts with classifying the raw data (composed of time stamp, screen *x* coordinate, screen *y* coordinate, and often pupil size) into eye-movement events such as fixations, saccades, and blinks. One approach is to manually classify eye-movement events, for instance by labeling stable periods in time series plots of the recorded horizontal and vertical gaze position data as fixations. Instead, to efficiently obtain reproducible classification results using a describable (Hooge et al., 2018) process, many studies use an existing manufacturer-provided or custom-made event detection algorithm to do such classification. Algorithms often use thresholds of certain eye-movement parameters (e.g., movement dimension, velocity, or duration) to detect and classify the events in the data. Algorithms differ in the details of their classification strategy, such as which eye-movement event is classified first or what threshold settings are used, yet many commonly used algorithms use predefined thresholds that are based on the eye-movement characteristics of human adults.

#### Effects of data loss and lower quality data on eye-movement event classification

A few dog eye-tracking studies have demonstrated that a large amount of data loss can make eye-movement event classification by means of algorithms impossible. For example, Kis et al., (2017) and Gergely et al., (2019) reported that the amount of recorded data per trial in dogs was too little to apply an eye-movement event classification algorithm, and for this reason, they only counted the number of data samples (referred to as gaze or look in the studies) inside their AOIs instead of number or (total) duration of fixations or saccades that are more typical variables in eye-tracking studies (Table 1). While this is an extreme example demonstrating the impact of data loss, even if classification using an algorithm is possible, high noise levels and frequent gaps in data may significantly affect the detection of eye-movement events. When algorithms that are not robust to these two issues are used to process such lower quality data, the algorithm outputs contain more artefacts, i.e., physiologically impossible eye movements, such as too short fixations and saccades (Holmqvist et al., 2012). Moreover, the lower the quality of the data, the more differences there are between different algorithms and thresholds settings in their outputs. For example, Wass et al., (2013) demonstrated that when they used a system built-in (dispersion-based) algorithm with default thresholds to classify data of infants, the eye-movement event results were heavily influenced by inter-individual variations in data quality. Using their custom-made algorithm that could further identify and eliminate artefactual fixations, fixations could be identified more reliably (Wass et al., 2013). Such differences in outputs between the event-detection algorithms were however not observed when classifying high-quality adult data, suggesting that the classification of lower quality data is more sensitive to the choice of algorithm. Due to this reason, in human eye-tracking practice, a greater caution has long been advised for classifying eye-movement events if the quality of data is lower (Hessels et al., 2017).

#### Effects of species-typical eye-movement characteristics on eye-movement event classification

Only a few studies have compared eye movements of humans and non-human animals (e.g., Blount, 1927; Martinez-Conde and Macknik 2008), making it all the more important to ask whether and how eye-movement characteristics of animals may differ from those of human adults, and how this may further complicate the choice of threshold settings in event classification algorithms. In dogs, using a custom-made velocity-based algorithm (Nyström & Holmqvist, 2010) that can tailor the velocity thresholds based on the noise level of the data, Park et al., (2020) found that saccades of dogs are slower and fixations of dogs were on average around four times longer than those of humans. Based on these findings, they suggested that when fixation classification is performed on dog eye-movement data using system built-in dispersion-based algorithms, the default minimum fixation duration threshold of these algorithms should be adjusted to a higher value, since the default settings are likely based on the fixation behavior of human participants. Besides, whether the amount of missing data and inaccuracy in dogs is further exacerbated by possibly different blink characteristics from those of humans has not been explored. Blinks create not only gaps in the recorded gaze data but also artefacts around their start and end. If, for example, a dog blink is much longer or more frequent than human blink, their contribution to the data quality probably should not be overlooked.

### Goal of the study

The above overview reveals that dog eye-tracking data are often of lower quality, for instance possibly due to their unique morphology that is not fully compatible with current P–CR systems. Furthermore, the eye-movement characteristics of dogs that are crucial to event classification differ from those of humans. Methodological studies of human eye-tracking data lead us to expect that this complicates the analysis of dog data and needs to be taken into account. The current investigation had three objectives, 1) to know how morphological characteristics unique to dogs could interfere with the tracking performance of P–CR eye trackers, and 2) to compare another eye-movement event, blinks, of dogs and humans to examine its potential consequences on data quality, and 3) to examine how the lower quality of dog data may differentially affect eye-movement event classification by comparing how sensitive the event classification outputs and central measures derived from the outputs are to the choice of algorithm. For this aim, we have tested dogs and humans in an eye-tracking experiment where they viewed a set of pictures of human faces, dog faces, and control objects, similar to previous dog eye-tracking studies.

First, to examine how the vision-related morphological characteristics of the eyes and facial regions of dogs may lead to tracking loss in a P–CR eye-tracking system and to lower data quality, we have used a P–CR system that can show a live view of a tracked eye. Second, in order to investigate whether dogs and humans show differences in their blinks, we have compared the frequency and duration of blinks between dogs and humans. Finally, we have compared the fixation classification output (proportion of artefactual fixations and fixation duration) as well as three commonly used fixation-related outcome variables (time to first fixation, total fixation count, and total fixation duration) between our dog and human data using fixations identified by two algorithms (the built-in algorithm of the EyeLink eye-tracking system and a custom implementation of the Nyström and Holmqvist (2010) algorithm). As further measures of noise level in the data, we have also compared the stability of human and dog fixations classified by Nyström and Holmqvist (2010) algorithm as well as the velocity thresholds that this algorithm ended up using for each trial (as this algorithm adopts higher thresholds for noisier data, in contrast to the EyeLink algorithm which uses a single fixed threshold).

While the overall quality of dog data is likely poorer than that of human adults, the worst quality can be avoided through careful selection and training of subjects, and well-performing dog subjects can yield data of the quality comparable to that of humans. On the other hand, for experimental paradigms that use tasks not crucially dependent on the quality of data (e.g., preferential looking task), many dogs would likely deliver good enough data. Importantly, based on our findings we suggest practical solutions and recommendations that future dog eye-tracking studies could adopt to improve the quality of their recordings and data analysis.

## Method

The eye-movement data analyzed in the current paper are part of a larger dataset comprising an experimental study comparing human and dog face viewing behavior (in preparation). This data was also part of the dataset previously used for Park et al., (2020).

### Subjects

We recruited dog subjects by contacting dog owners from the Vienna area who had previously agreed on participating in behavioral and cognitive studies in the Clever Dog Lab. Initially, 33 dogs were recruited for pre-experiment training. Of these, eight dogs had vision-related morphological characteristics that interfered with the ability of the eye-tracking system to track them. We collected eye-video images from these dogs to examine the implications of the morphology, yet the dogs were excluded from further data collection. A further ten dogs could not complete the pre-experiment training, thus the sample with which we started data collection consisted of 15 dogs. 14 dogs could complete all trials, and the data of these 14 dogs were analyzed (age M = 5.57 years, SD = 2.88 years; sex: six males and eight females). The dog subjects were one Akita Inu, four Border Collies, one Boxer, one Petit Brabancon, one Golden Retriever, two Siberian Huskies, one Jack Russell Terrier, one Parson Russell Terrier, one Rhodesian Ridgeback, and one mixed breed. In addition, 15 human participants (age M = 29.2 years, SD = 10.5 years; gender: six males and nine females), volunteering graduate students, dog owners or university staff with normal or near normal vision without glasses (glasses were off during the experiment), completed all trials. The human subjects did not present with droopy eyelids or other morphological characteristics that might have interfered with the eye-tracker’s performance.

### Ethical statement

All experimental protocols were approved by the institutional ethics and animal welfare committees of the University of Veterinary Medicine, Vienna and Medical University of Vienna in accordance with the GSP guidelines and national legislation (02/03/97/2013 and 1336/2013, respectively). All methods were carried out in accordance with the relevant regulations and guidelines. Informed consent was obtained from all human participants and dog owners before the study was conducted.

### Stimuli

Each subject was shown 24 images from three different categories (human face, dog face, and control object) in either an upright or an inverted orientation, for a total of 24 trials. The stimuli were originally designed for another study (Park et al. in prep.). The images were generated using the freely available image processing tool GIMP or collected from the Radboud FACE Database (Langner et al., 2010) and Internet websites with their permission. The sizes of the images ranged from 246 to 411 pixels in width and 257 to 341 pixels in height which corresponded to angular widths and heights of approximately 8-12^∘^ and 7-10^∘^, respectively. For each trial, one image was presented on the left or right side of the screen, where the closer edge of an image was 100 pixels (3^∘^) horizontally away from the center of the screen. Stimuli were presented in random order with presentation side balanced. The stimulus presentation was controlled by an executable file generated by the Experiment Builder (version 1.10.165) of SR Research.

### Apparatus: EyeLink 1000 and its eye video

**Fig. 1:**
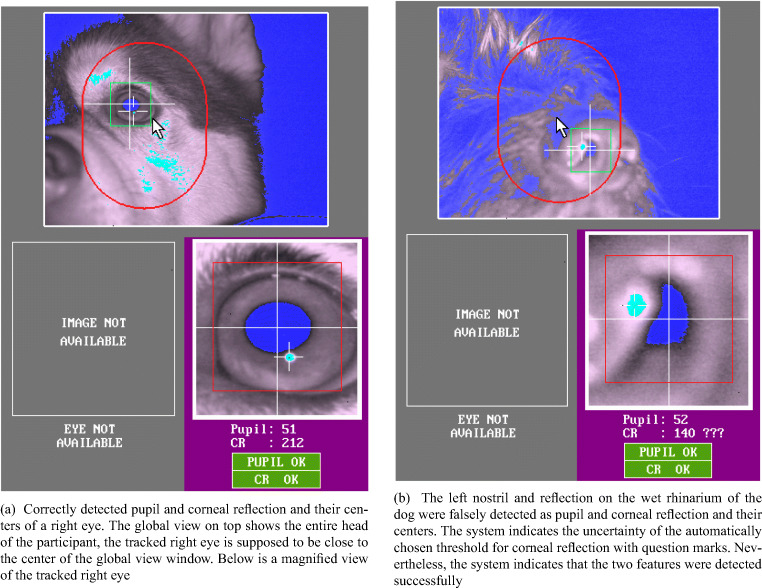
Examples of correct (**a**) versus false (**b**) detection of pupil and corneal reflection

Both dogs and humans were tested at the Clever Dog Lab, using the same setup and an EyeLink 1000 eye tracker (SR Research) with 35-mm camera lens in desktop. The gaze position of the right eye was recorded at 1000 Hz from a distance of 50–55 cm to the eye. The pupil was detected using centroid mode. The eye video, provided by the system by default, is a live view of the eye-tracking camera with its lens facing the eye of the subjects. In the live view, the recorded eye is overlaid with markings of the detected pupil and corneal reflection and their centers, as shown in Fig. 1. While tracking each subject’s eye, the experimenter used the eye-video view to observe the participant’s eye and surrounding ophthalmic structures in detail, to examine if they interfere with tracking of the pupil and corneal reflection centers. When interference occurred, we made screenshots of the eye-video images for later examination. Some of these screenshots are presented in the results section.

### Experimental procedure

Before the recording, each dog was trained for staying still and looking straight at the screen, and also to look at calibration points (for the details of the training, see Park et al. 2020). Human participants were not trained, but simply instructed to refrain from head movements. At the start of each experiment, we calibrated the right eye of each participant until a subsequent validation yielded an average error less than 1.5^∘^. Most human subjects did not require repetition of the calibration and validation procedures, yet for most dog subjects we needed to repeat the procedures at least one more time. Each trial started with a display containing a fixation point at the center of the screen. After the experimenter confirmed the participant looked at the fixation point, a trial stimulus was presented for seven seconds, after which the next trial started. After each block of two trials, the dog or human participants could move or eat a food reward. The calibration procedure was performed before every block, and also before the next trial if there was obvious movement (head rotated or out of the chinrest). The average accuracy obtained during the training and the experiment was 0.88^∘^ for dogs and 0.51^∘^ for humans. Eye-movement recordings were stored to file for offline analysis.

### Eye-movement event classification

We used two different eye-movement event classification algorithms to process the raw eye-movement data: the built-in algorithm of the EyeLink 1000 from SR Research and the custom Nyström and Holmqvist (2010) algorithm as implemented by Niehorster et al., (2015) (available at https://github.com/dcnieho/NystromHolmqvist2010). To extract the fixations and blinks classified by the EyeLink algorithm, we used Data Viewer (version 1.11.1) of SR research. Classification of fixations and blinks through the Nyström and Holmqvist (2010) algorithm was done in MATLAB (MathWorks, Natick, MA, USA). Both algorithms compute instantaneous velocity for each data sample and then compare a velocity threshold to the resulting gaze velocity signal to determine where saccades occurred. Somewhat simplified, both algorithms classify samples above their velocity threshold as saccades, whilst the rest of the data below the threshold is further processed to yield classification as fixations, blinks, and possibly other events. The important difference between the two algorithms lies in how they determine the velocity threshold. In our study, a fixed saccade velocity threshold of 22^∘^/s was used for the EyeLink algorithm, which corresponds to the system’s “psychophysical experiment” configuration. In contrast, the Nyström and Holmqvist (2010) algorithm does not use a single pre-determined velocity threshold for saccade classification. Instead, the algorithm requires users to set only a high initial velocity threshold, which is then refined by the algorithm for each trial based on the level of noise within that trial. The final velocity threshold for a trial is thus adapted to the data quality of the trial, and will vary between trials. The initial velocity threshold we used for the Nyström and Holmqvist (2010) algorithm in this study was 40^∘^/s. How blinks are classified also differs between the two algorithms. For more details of each algorithm, the reader is referred to the EyeLink 1000 user manual (version 1.5.2) and Nyström and Holmqvist (2010).

### Statistical analysis

For all statistical tests, we used (generalized or general linear) mixed-effects models, in order to take into account the baseline differences between different individuals (Bolker et al., 2009). The specifications of the statistical models are described in Table 3. An *α*-level of 0.05 was used to judge the significance of the model parameters (type III ANOVA using Wald Chi-square tests), of the difference in the estimated means (two-tailed Z or *t* tests), of pairwise comparisons (Lenth & et al., 2016) or of the difference between the distribution functions of the data (two-sample Kolmogorov–Smirnov and Wilcoxon tests). Each *P* value is presented with a corresponding surprisal (Shannon information) value ($S = -\log _{} P$) that signifies the amount of evidence supporting the result of the *P* value in terms of how often one gets all heads for the same amount of coin flip (e.g., For *P* = 0.05, *S* = 4.32 which corresponds to having four heads on four times of coin tosses. For more information, see Rafi & Greenland, 2020). In case of multiple pairwise comparisons results, *P* values were adjusted using multivariate *t*-adjustment. All tests were conducted in R (R Core Team, 2019) version 4.0.0 using the lme4, car, effect, emmeans, MuMIn, glmmTMB, and stats packages. The data and R scripts used for statistical analysis are available at https://zenodo.org/deposit/4783359. Plots visualizing the results are presented with goodness of fit statistics of the tested models (${R_{m}^{2}}$ = marginal *R*^2^, ${R_{c}^{2}}$= conditional *R*^2^. S = standard deviation of the residuals) and significance code: 0 < ‘***’ < 0.001 < ‘**’ < 0.01< ‘*’< 0.05.

**Table 2:** Comparisons of relevant morphological characteristics of the eyes and eye region across dogs, non-human primates, and humans

Vision-related/	Dogs	Non-human primates	Humans	References
ophthalmic morphology				
Snout	shape varies by breed^1^	non-existent	non-existent	1. Roberts et al., (2010)
Eye position in skull	20 ^∘^ lateral on average^1,2^	forward-facing	forward-facing	1. McGreevy et al., (2004)
	laterality varies by breed			2. Miller and Murphy (1995)
Eye cleft	circular	circular^1^	horizontally elongated^1^	1. Kobayashi & Kohshima (1997, 2001)
3rd eyelid	dark-pigmented or pale pink ^2,3^	rarely existent ^1^	non-existent ^1^	1. Butler and Hodos (2005)
				2. Barnett (1978)
				3. Maggs et al., (2013)
Eyelid margins	mostly dark-pigmented ^1^	mostly dark-pigmented	reddish white	1. Petersen-Jones and Crispin (2002)
Sclera color	white^2^	white to mostly dark-pigmented^1^	white^1^	1. Kobayashi & Kohshima (1997, 2001)
				2. Petersen-Jones and Crispin (2002)
Sclera visibility	mostly invisible	visible in some species^1^	highly visible^1^	1. Kobayashi & Kohshima (1997, 2001)

Corresponding references for each table cell content are indicated with superscripts

**Table 3:** Specifications of statistical models tested in the study

Analysis	Model	Model specification (in Wilkinson notation)		family	link function
Blinks	Blink count	count $\sim $	algorithm ∗ species + (1 + name)	Poisson	log
	Duration of a blink	duration (ms) $\sim $	algorithm ∗ species + (1 + name)	Gamma	log
	Proportion of	proportion $\sim $	algorithm ∗ species + (1 + name),	Binomial	logit
Implications of	artefactual fixations		weights = total fixation count		
data characteristics	Duration of a fixation	duration (ms) $\sim $	algorithm ∗ species + (1 + name)	Gamma	log
	Fixation stability	BCEA (*pixels*^2^) $\sim $	species + (1 + name)	Gamma	log
	Velocity threshold	trial velocity threshold (deg/s)$\sim $	species + (1 + name)	Gaussian	identity
Three fixation-related	Time to first fixation	latency(ms) $\sim $	algorithm ∗ species + (1 + name)	Gamma	log
dependent variables	Total fixation count	count $\sim $	algorithm ∗ species + (1 + name)	Poisson	log
	Total fixation duration	total duration (ms) $\sim $	algorithm ∗ species + (1 + name)	Gamma	log

#### Analysis of blinks

To compare the blinks of dogs and humans, we used the blinks output by both algorithms, and statistically tested the difference between algorithms and species. Blinks shorter than 80 ms were considered artefacts (e.g., data loss due to other reasons) and excluded from the statistical analysis.

#### Analyses to examine the implications of different data characteristics between species

##### Artefactual fixations, fixation duration and stability, and trial velocity thresholds

First, we have examined the frequency of artefactual fixations identified by both algorithms when run on both dog and human data. For this analysis, we defined (non-artefactual) fixations as episodes of gaze data where the participant looked at the same location on the screen for at least 50 ms (Hessels et al., 2018). Accordingly, the frequency of artefactual fixations was operationalized as the percentage of classified fixations shorter than 50 ms. By default, the Nyström and Holmqvist (2010) algorithm removes fixations with a duration below 50 ms. This functionality was switched off to enable us to observe the artefactual fixations produced by the algorithm. We statistically tested whether both algorithms produced larger proportion of artefactual fixations when classifying dog than human data, and whether the difference in the proportions between algorithms is greater for dog than human data. Fixations, either artefactual or non-artefactual, that were not on the stimuli were excluded from the analysis. We further examined whether the difference between the algorithms in reported duration of non-artefactual fixations was larger for the dog than the human data. To do so, the artefactual fixations were excluded. We statistically tested the difference between species in the estimated mean and distribution of duration of fixations classified by each algorithm.

Furthermore, we ran two analyses on two additional measures derived only by the Nyström and Holmqvist (2010) algorithm in order to make further comparisons on how similarly or differently the Nyström and Holmqvist (2010) algorithm classified dog and human data. The first measure was the bivariate contour ellipse area (BCEA) measure of the fixations classified by the Nyström and Holmqvist (2010) algorithm. BCEA of a fixation is the size of an elliptical area ((^∘^)^2^) that encompasses 68% of the data samples belonging to a fixation (for further details of its calculation and usage, see Crossland et al., (2002), Holmqvist et al., (2011, chap 11.26), and Niehorster et al.,, (2020b)). The smaller the BCEA of a fixation is, the more stable or less dispersed the fixation is. We statistically tested whether the estimated mean BCEA value of dog fixations is larger than that of human fixations. The second measure was the velocity thresholds the Nyström and Holmqvist (2010) algorithm used for each trial to classify the data. We statistically tested the difference between species in the estimated mean velocity threshold across trials and the distribution of the velocity thresholds. The amount of data included in the data analysis is shown in Table 4.

**Table 4:** The amount of data used in the analysis of lower data quality implications and the analysis of common fixation-related dependent variables

Analysis	Model	Algorithm	Dog	Human	Data unit
Blinks	Duration of a blink	EyeLink	180	433	Number of
		Nyström and Holmqvist (2010)	220	502	blinks
	Duration of a fixation	EyeLink	1268	5212	Number of fixations
Implications of		Nyström and Holmqvist (2010)	940	4772	
data characteristics	BCEA	Nyström and Holmqvist (2010)	940	4772	Number of fixations
	Velocity Threshold	Nyström and Holmqvist (2010)	335, 93%	360, 100%	Number of trials, percentage (out of 360 trials)
	Time to first fixation	EyeLink	303, 84%	360, 100%	
		Nyström and Holmqvist (2010)	287, 80%	360, 100%	
Three fixation-related	Total fixation count	EyeLink	303, 84%	360, 100%	Number of trials,
variables		Nyström and Holmqvist (2010)	295, 82%	360, 100%	percentage (out of 360 trials)
	Total fixation duration	EyeLink	303, 84%	360, 100%	
		Nyström and Holmqvist (2010)	295, 82%	360, 100%	

##### Three common fixation-related dependent variables

Besides fixation duration, many eye-tracking studies also report one or multiple of the following three fixation-related dependent variables: time to first fixation, total fixation count, and total fixation duration. They are directly influenced by the result of fixation classification that, as addressed by the previous analyses, is dependent on how well the applied algorithms cope with lower data quality and eye-movement characteristics different to those of human adults. Therefore, we have examined the effects of species, choice of algorithm and their interaction for these fixation variables. For this analysis, trials that had no fixation on the stimulus were excluded, and also artefactual fixations were excluded in the calculations of total fixation count or total fixation duration. Time to first fixation is the measure of duration subjects took to make their first fixation on the stimulus in each trial. Eight trials of dogs were further excluded from the analysis of this variable, because the dogs in these trials had been fixating on the stimulus area before the stimulus appeared. Total fixation count and total fixation duration are the count and duration measures of all fixations made on the stimulus in each trial, respectively. The amount of data included in the analysis is shown in Table 4.

## Results

### Dog-unique morphology interferes with pupil and corneal reflection detection performance of a P–CR system

We have observed that certain morphological characteristics of dogs interfere with the performance of a P–CR system, mostly by blocking the view of the pupil and corneal reflection or interfering with reliable determination of their centers. In this section, we describe in detail how various morphological characteristics interfere with eye tracking, and present example eye-video images collected from some of the eight dogs that were excluded from data collection. For comparison, Fig. 1a shows the example of properly detected pupil and corneal reflection and their centers in a dog without problematic morphological characteristics. Note that for all example eye-video images, the eye on the right side of the images is the right eye of a dog.

#### Snout

Unlike humans, dogs possess a snout or muzzle, which typically includes their nose, mouth, and parts of the upper and lower jaws. The tip of the muzzle is the rhinarium, a non-hairy nose surface around the nostrils which is kept wet in most healthy dogs (Kröger & Goiricelaya, 2017). The width and length of the snout and the eye laterality (position of the eyes in the skull) vary extensively across breeds. In brachycephalic (short-nosed) dogs, such as the pug, the snout is almost absent, but in dolichocephalic (long-nosed) dogs, such as the grey hound, it is long and thin (McGreevy et al., 2004; Roberts et al., 2010). We have observed that long snouts may block the view of the eye-tracking camera, especially when the dog moves its head slightly backwards or upwards in the chinrest. Such blocking of the camera view made the system lose track of the pupil or corneal reflection. In rare occasions, a nostril and the wet surface of the rhinarium could be falsely detected as the pupil and corneal reflection, if the dog in the chinrest moves even further backwards. Surprisingly, the system still indicated successful detection of their centers (Fig. 1b).

#### Eye laterality

While the eyes of most humans and non-human primates are forward-facing, the eyes of dogs are located in their skull with different laterality (Here, we use the term laterality to refer to the location of the eyes in the skull i.e., how laterally the eyes are positioned relative to the sagittal plane of the skull). The eyes of mesocephalic dogs have an average laterality of 20^∘^ (Miller & Murphy, 1995), and those of brachycephalic dogs are more laterally directed. Even within mesocephalic dogs, each dog breed or individual has slightly different laterality of the eyes. To adjust to this, we needed to set the camera angle (the angular orientation of the eye-tracker camera relative to the horizontal and vertical plane on which it sits) for each dog at the beginning of and also during sessions, so that the camera lens faces the right eye of a dog optimally. This was checked by monitoring the eye-video output. If such adjustments happened during a session, e.g., due to slight head movement, we ran additional calibrations. These adjustments had to be made much more frequently than for human participants, for whom a given setup could be easily used for another individual without further adjustment.

#### Circular and small eye cleft

**Fig. 2:**
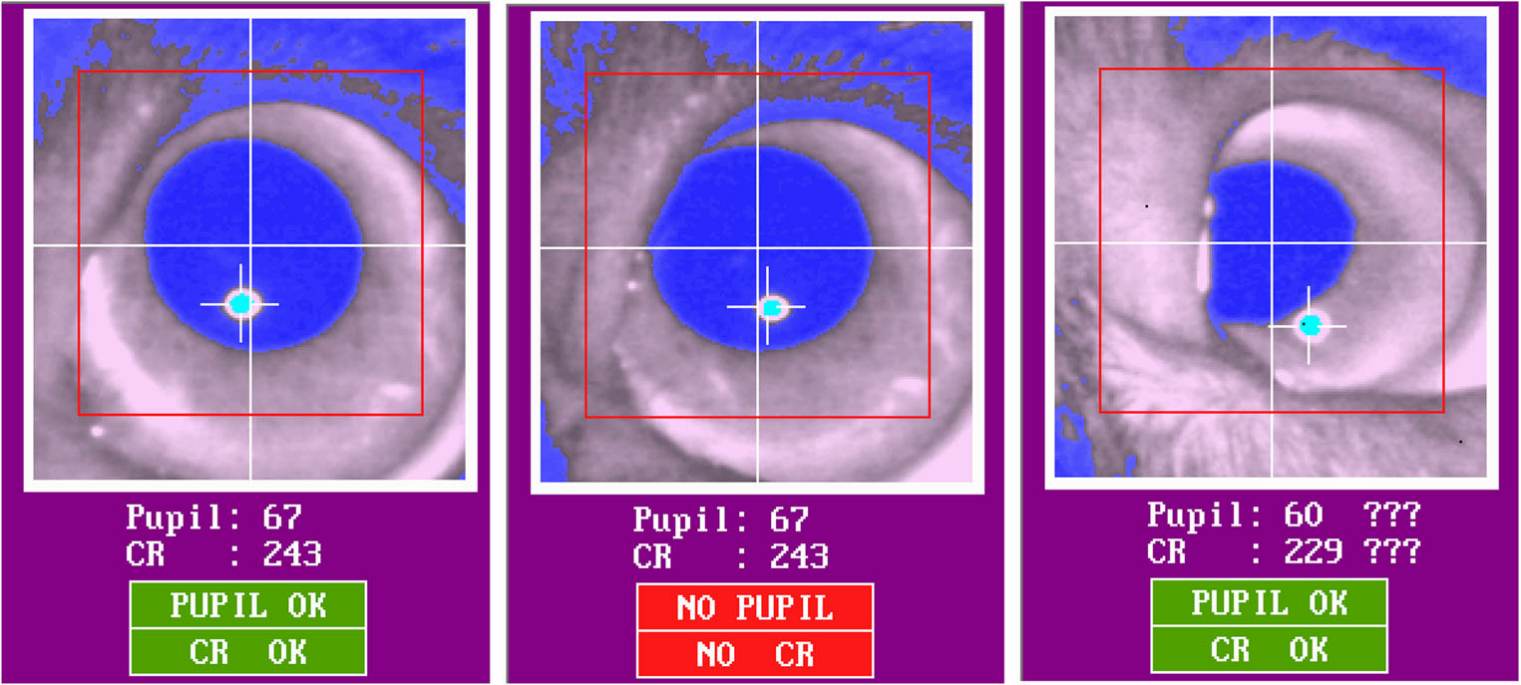
The left eye-video image shows correctly detected pupil and corneal reflection centers. In the image in the middle, the upper part of the pupil is slightly covered by the nasal part of the upper eyelid. Despite that the pupil center and corneal reflection are indicated, the system indicates a failure of pupil and corneal reflection detection. In the right image, the left part of the pupil is covered by the nasal part of the upper eyelid. The auto-threshold algorithm indicates the uncertainty of the chosen threshold values by means of the question marks

**Fig. 3:**
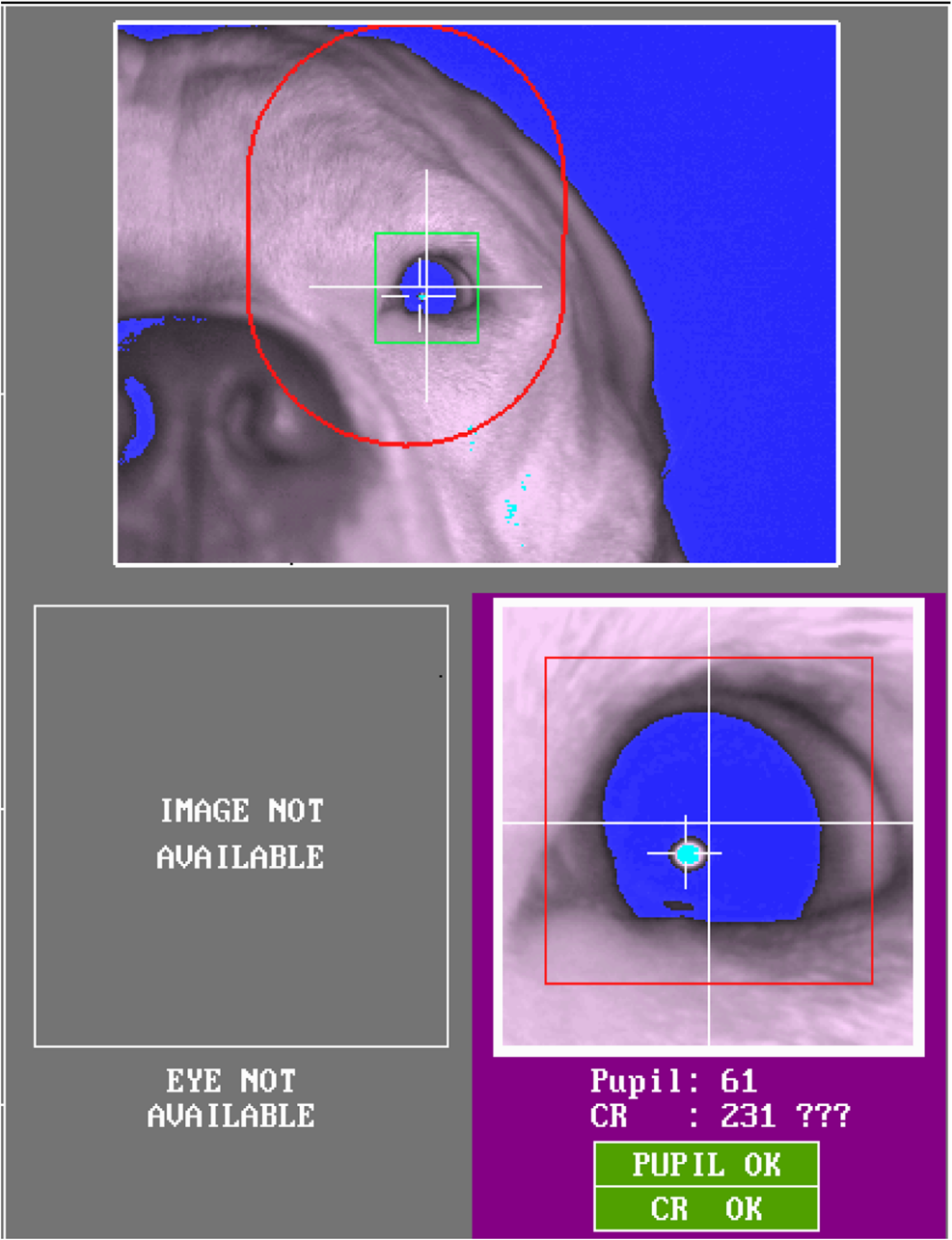
The pupil of a dog with relatively small eye cleft is covered by the lower eyelid

The extent to which the pupil can be tracked by a P–CR system highly depends on the shape of participants’ eye cleft (palpebral fissure). In both dogs and humans, there are individuals with droopy upper eyelids, characterized by relatively low height of the opening between the eyelids. In those individuals, as shown in the middle and right images in Fig. 2, their upper eyelid can cover part of the pupil, leading to misdetection of the pupil center by the system. Even though the system might indicate successful detection of the partly blocked pupil and its center as shown in the right image of Fig. 2, the calculated pupil center is incorrect, because its calculation is based not on the entire, but on a part of the pupil. This consequently introduces an offset in the estimated gaze location (Holmqvist et al., 2011). Importantly, we have observed that the dog eye cleft is more likely to produce such pupil coverage than that of humans. The eye cleft of dogs is circular as in most non-human animal species. Humans, on the other hand, have a unique eye cleft that is elongated in the horizontal direction. This provides relatively more freedom for the human eye balls to move sideways without having part of the pupil covered by the eyelids (Kobayashi & Kohshima 1997, 2001). The circular eye cleft of dogs lacks such spatial freedom as shown in the right image of Fig. 2 where the nasal part of the upper eyelid is blocking part of the pupil. Also, the pupil of dogs seems to cover a relatively large area of the eye, likely because the size of their eye cleft is smaller than that of humans (we were unable to find a comparison of pupil size between humans and dogs). In individuals having particularly small eye cleft, the pupil could be covered even by their lower eyelid (Fig. 3). However, we cannot exclude the possibility that the detected pupil area of the individual dog in Fig. 3 is actually bigger than that of other dogs, either because the pupil is actually bigger, or because part of the iris is erroneously included in the pupil area.

#### Third eyelid and eye mucus on the cornea

**Fig. 4:**
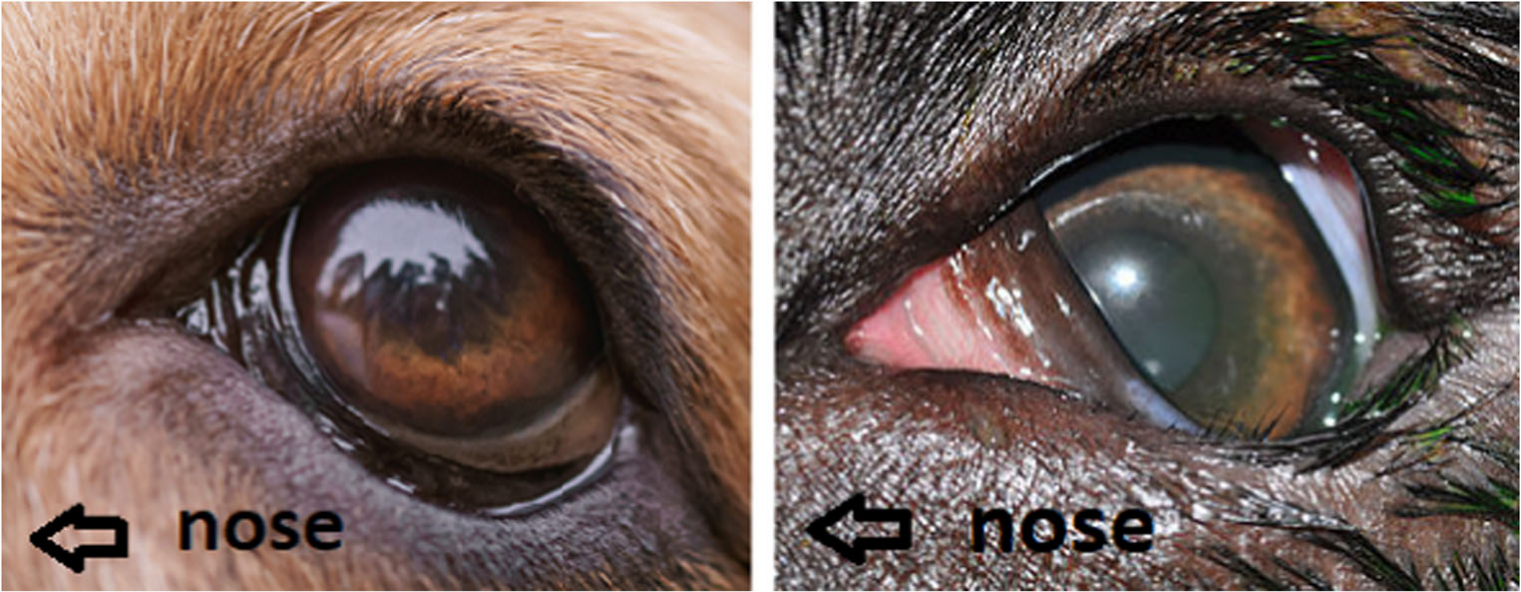
Each image shows a left eye of a dog with a third eyelid and dark eyelid margins. The *left image* shows a typical example of a third eyelid that can be seen in most dogs where only the dark brown outer edge of the third eyelid is visible in the nasal corner. However, in some dogs, as in the dog of the *right image*, not only the dark brown outer edge but also the inner part of the third eyelid in light salmon color is visible which can also cover part of the pupil. Note that the eyelid margins of both dogs are dark brown. Photo/Image courtesy: the first image is acquired royalty free from www.shutterstock.com. The second image is reproduced with permission from Dr. Noelle McNabb at www.animalvisioncare.com

**Fig. 5:**
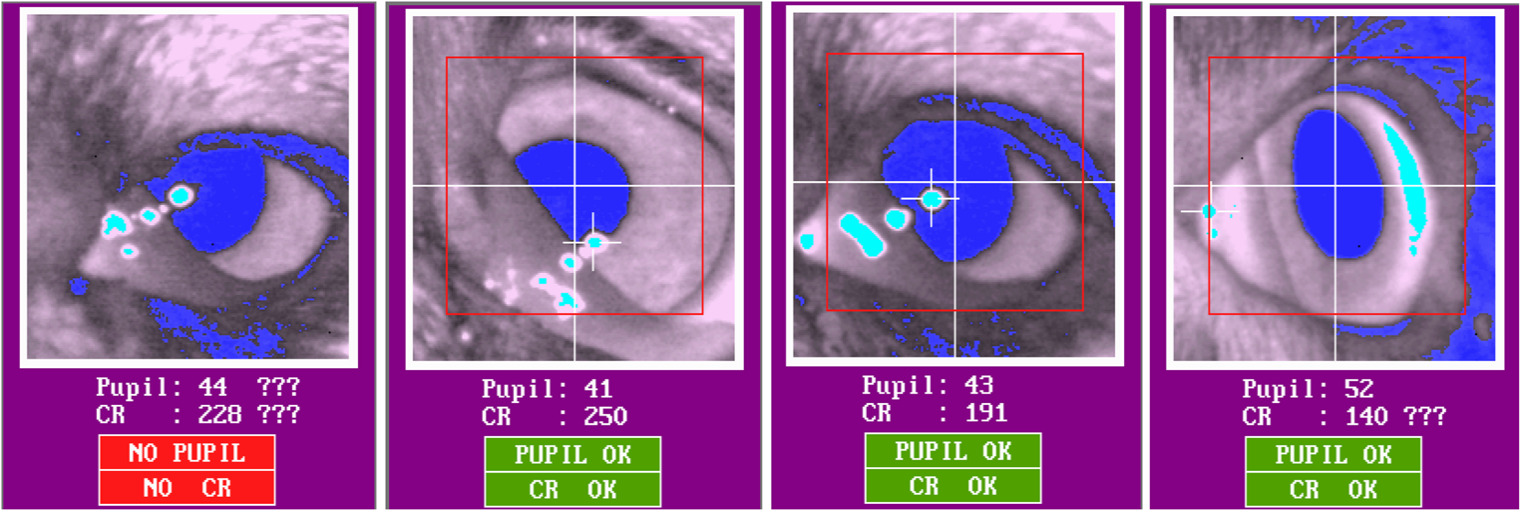
In the first three images, the lower nasal part of the pupil is covered by the third eyelid. There are three extra infrared light reflections in diverse sizes. In the fourth image, when the gaze of the dog is more temporally displaced, the reflection on the third eyelid is detected as a corneal reflection, while an elongated reflection appears on the other side of the cornea

Another characteristic unique to dogs that can cause blockage of the pupil is the third eyelid (i.e., nictitating membrane) (Fig. 4). The third eyelid is dark-brown and located in the inferior conjunctival sac, between the cornea and the lower eyelid. As shown in the first image of Fig. 4, in most dogs, it briefly appears when they blink and much of it disappears when the blink ends leaving only a very small part (outer edge) of it visible near the nasal corner of the eyelids. When the third eyelid is inflamed, the condition called ‘cherry eye’, it is highly visible without blinking as it gets swollen, red, and protrudes out. However, in a few dogs we have observed that the third eyelid is highly visible without blinking or obvious inflammatory conditions. Such highly visible third eyelid could cover part of the pupil as shown in the second image of Fig. 4. Similarly to what happens with the droopy upper eyelid or small eye cleft, the covered pupil can either lead to track loss or to misdetected pupil center location as in Fig. 5.

Moreover, the third eyelid could interfere with the detection of corneal reflection. As a gland, the surface of the third eyelid is normally wet and covered with mucus (Maggs et al., 2013; Petersen-Jones & Crispin, 2002). As the wet surface on the third eyelid is typically reflective, one or several thresholded reflections may appear on the third eyelid. The first three images in Fig. 5 demonstrate the extra reflections on the third eyelid. Such extra reflections

may be potentially misidentified by the system as the real cornea reflection and become the source of artefactual eye movements (Holmqvist et al., 2011). On the fourth image of Fig. 5, where the viewing angle of the eye is extreme, the pupil is uncovered, yet the reflection on the third eyelid, on the left side of the image, is falsely detected as the corneal reflection.

#### Dark eyelid margins and hair

**Fig. 6:**
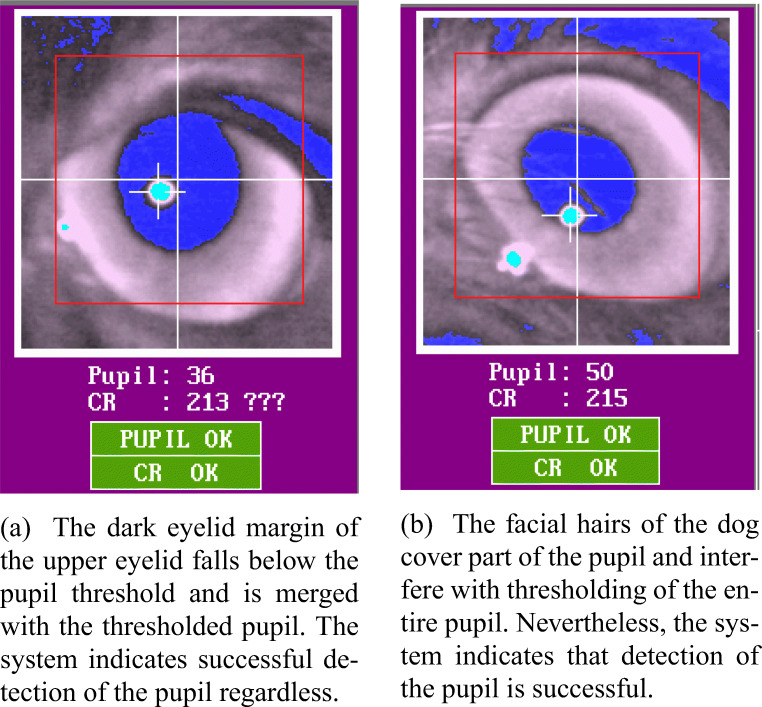
Eyelid identified as pupil (**a**) and hair covering the pupil (**b**)

In many dog breeds, such as the Border Collie and the Beagle, the eyelid margins are dark-colored and have much heavier pigmentation than that of other parts of their faces (Fig. 4). In contrast, the color of human eyelid is similar to that of other facial parts (Petersen-Jones & Crispin, 2002). As shown in Fig. 6a, we have observed that because of the dark color, the eyelid margins, especially the upper eyelid margin, of dogs could fall below the pupil threshold of the system, similarly to the pupil. Although the thresholded eyelid margin itself could be tolerated, when the distance between the pupil and the eyelid margin is small enough, more likely due to dog’s small eye cleft, they can become merged as shown in Fig. 5 (first and third images) and Fig. 6a. Such merging of the pupil and part of the eyelid margin often caused pupil detection failure. In some cases, the system indicated that the pupil was successfully detected despite the merging (Fig. 6a). However, similarly to the blocked pupil, the pupil center data based on the pupil merged with the eyelid margin is incorrect and causes an offset in the estimated gaze location. Similar problems have been reported in human studies, for instance when subjects wear dark eye makeup products, such as eyeliner or mascara (Nyström et al., 2013).

Dogs usually have eyelashes pointing upward or downward in upper or lower eyelid margins, respectively. Due to their outward directions, the eyelashes usually do not interfere with detection of the pupil or cornea reflection in dogs. However, dogs have hair of similar thickness also on their nose, and other areas near their eyes. In some dogs, thick and rigid hair on their nose or the downward pointing hair on their forehead interrupted the view of the camera as shown in Fig. 6b. These pieces of hair lead to incomplete thresholding of the pupil which causes an offset in the estimated gaze location.

#### Other conditions

**Fig. 7:**
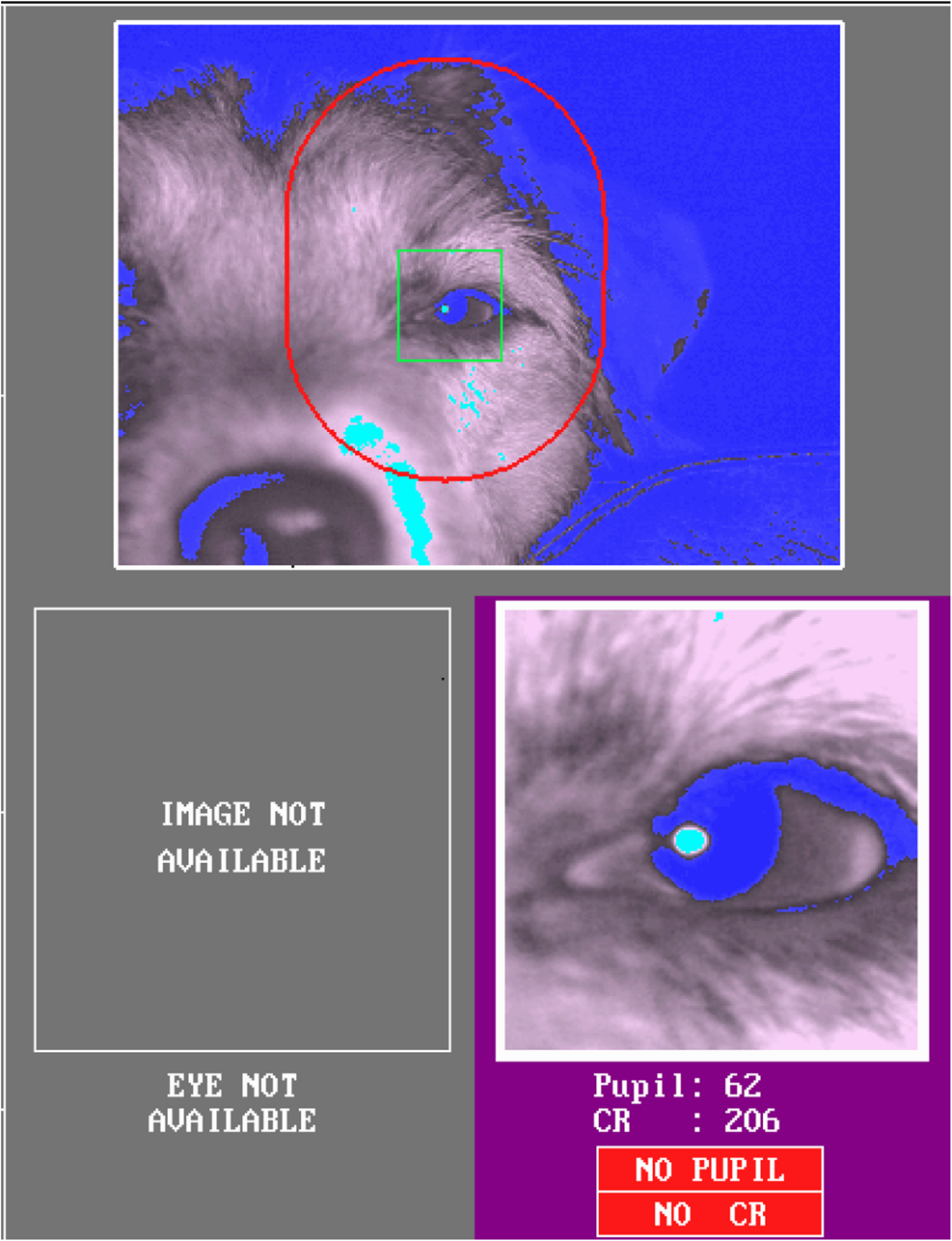
The dog eye is partially closed due to decreased arousal induced by exhaustion and hot weather. The system indicates that the pupil and corneal reflection are not detected

There are also certain ophthalmic conditions which can cause pupil blockage similarly to droopy upper eyelids. Lagophthalmos is a condition, in which the eye cannot be fully closed (Carrington et al., 1987; Nakajima et al., 2011). It is more prevalent in some brachycephalic dog breeds which have a shallow eye orbit (Gelatt, 2018). While complete closing of the eyes, blinking, is effectively detected by most current eye-tracking system algorithms, partial blinking is not. Partial blinks appear as sudden downward eye movement in the eye-tracking data and can be falsely detected as saccades. Additionally, as shown in Fig. 7, we have observed that decrease in the level of attention or alertness in dogs also causes partial eyelid closure as similarly observed in humans (Dasgupta et al., 2013). Such condition in dogs must be prevented if eye tracking is to be successful, yet recognizing it might be difficult if the eye tracker does not provide the eye video.

### Dogs blinked less often than humans, but their blink was longer

**Fig. 8:**
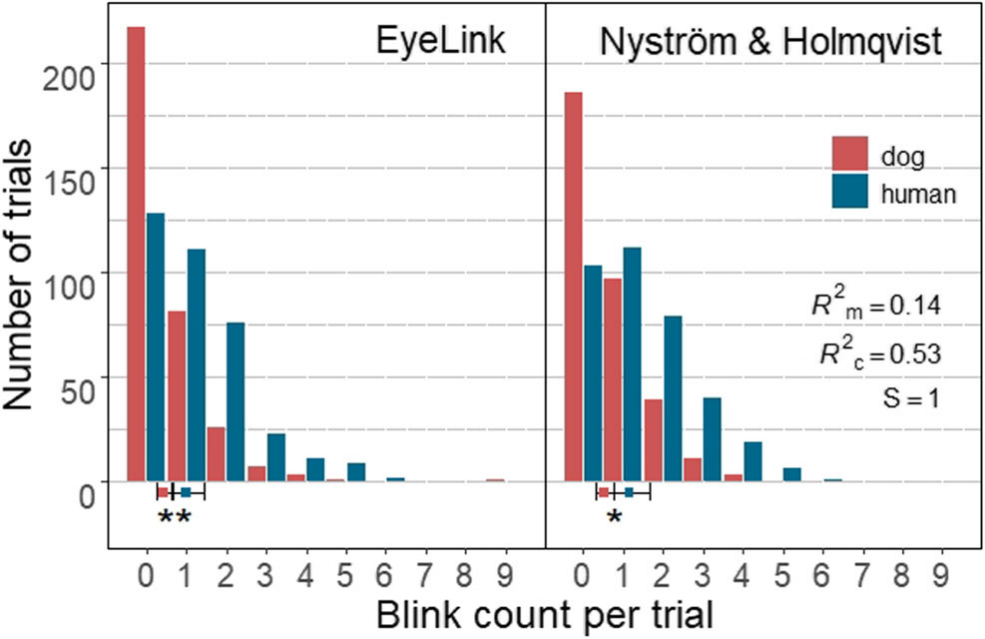
Each bar in the histograms represents the count of trials that had the given number of blinks denoted on the *x*-axis. *Solid square symbols* and *error bars* depict marginal means and 95% confidence intervals (± 1.96 ∗*SE*), respectively. Dogs on average blinked significantly less often than humans per trial

To quantify whether and how dogs and humans blink differently, potentially thereby affecting data quality, we have compared the rate (per minute) and duration of human and dog blinks classified by the two algorithms. As can be seen in Fig. 8, we found that independent of the algorithm used, dogs on average blinked significantly less frequently than humans per trial (EyeLink: Z = 3.01, *p* = .009, *S* = 6.8; Nyström and Holmqvist (2010): Z = 2.86, *p* = .015, *S*= 6.06). The estimated mean number of blinks per minute classified by the EyeLink algorithm was 3.43 (*SE* = 0.09) for dogs and 8.31 (*SE* = 0.20) for humans per minute, while for the Nyström and Holmqvist (2010) algorithm those were 4.11 (*SE* = 0.10) for dogs and 9.69 (*SE* = 0.23) for humans. However, the number of blinks between algorithms did not differ in either species.

**Fig. 9:**
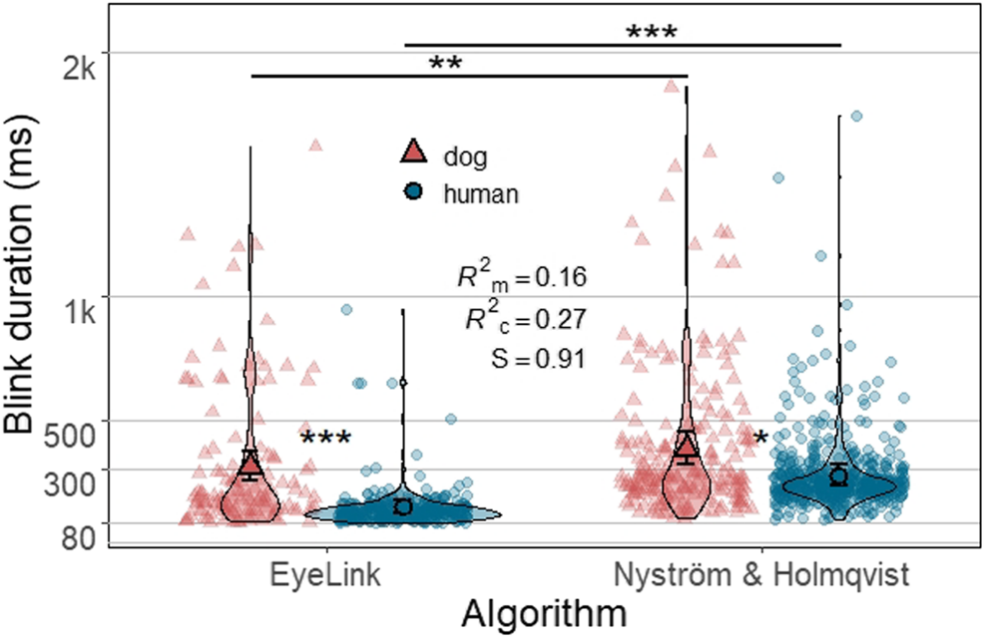
Each violin plot, demonstrating kernel probability density, depicts the distribution pattern of human or dog blink duration data classified by either algorithm. Note that blinks shorter than 80 ms are not included in the analysis. Nine data points with duration over 2000 ms are removed for better visualization of the remaining data. *Solid symbols* (a triangle or a circle) and *error bars* overlaid on the violin plots depict marginal means and 95% confidence intervals (± 1.96 ∗*SE*), respectively. *Background symbols* in softened color indicate data used in the model

On the other hand, the average duration of dog blinks classified by both algorithms was significantly longer than that of humans (EyeLink: Z = 5.89, *p* <.0001, *S* = 26.35; Nyström and Holmqvist (2010): Z = 2.68, *p* = .03, *S*= 5.06) (Fig. 9). The estimated mean duration of blinks classified by the EyeLink algorithm was 305 ms (*SE* = 29.8 ms) for dog and 143 ms (*SE* = 12.1 ms) for human blinks, while those of the Nyström and Holmqvist (2010) algorithm were 378 ms (*SE* = 33.9 ms) for dog and 274 ms (*SE* = 22.4 ms) for human blinks. Conversely, the estimated mean duration of both dog and human blinks differed significantly between the two algorithms, blinks classified by the EyeLink algorithm were significantly shorter than blinks classified by Nyström and Holmqvist (2010) (dog blinks: Z = 3.46, *p* = .002, *S* = 8.97; human blinks: Z = 16.38, *p* < .0001, *S* > 46.51). The algorithm effect size for human blinks was medium to large, while that of dog blinks was small (dog blinks: *d* = 0.24, 95% CI [0.10,0.37]; human blinks: *d* = 0.72, 95% CI [0.63,0.81]).

### Fixation classification and fixation-related measures are more susceptible to the choice of algorithm in dogs than in humans

#### Artefactual fixations

**Fig. 10:**
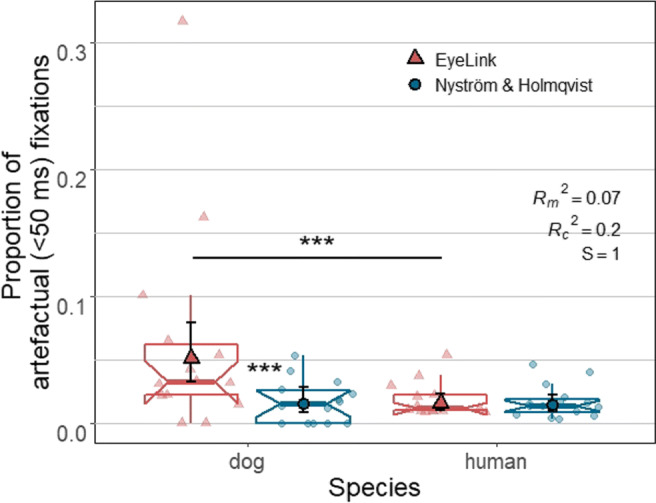
The lower and upper hinges of the notched box plots depict 25% and 75% inter-quantile ranges (IQRs) of dog and human data, respectively. The ranges of their upper and lower whiskers represent observations that belong within ± 1.5 * IQR from the hinges, respectively. Each notch of a box plot displays a confidence interval around the median (solid line in color matching each algorithm which is based on the median ± 1.58 * IQR/*n*. *Solid shapes* (a triangle or a circle) and *error bars* overlaid on the box plots depict marginal means and 95% confidence intervals (± 1.96 ∗*SE*), respectively. Individual data points (one data point per subject) are indicated by triangles and circles in softened color

Figure 10 shows the proportions of artefactual (< 50 ms) fixations in dog and human data classified by the two algorithms. Statistical tests showed that species, algorithm and also their interaction significantly affected the proportion of artefactual fixations in the algorithm output (interaction: X^2^(1) = 14.66, *p* < .0001, *S* = 12.92). The EyeLink algorithm produced a larger increase in artefactual fixations when classifying dog data than human data compared to the Nyström and Holmqvist (2010) algorithm. That is, although the proportion of artefactual fixations was higher for both algorithms when classifying dog data (X^2^(1) = 14.82, *p* < .0001, *S* = 13.04), pairwise comparison results showed that this difference between the species was significant only for the EyeLink algorithm (Z = 3.85, *p* = .0004, *S* = 11.29). On the other hand, across species the EyeLink algorithm produced a higher proportion of artefactual fixations than the Nyström and Holmqvist (2010) algorithm (X^2^(1) = 21.86, *p* < .0001, *S* = 18.38). However, pairwise comparisons showed that this difference between algorithms was only significant for dog data with effect size larger than one standard deviation (*Z* = 4.68, *p* < .0001, *S* = 16.55, *d* = 1.2, 95% CI [0.66, 1.78]).

#### Fixation duration and distribution of the duration

**Fig. 11:**
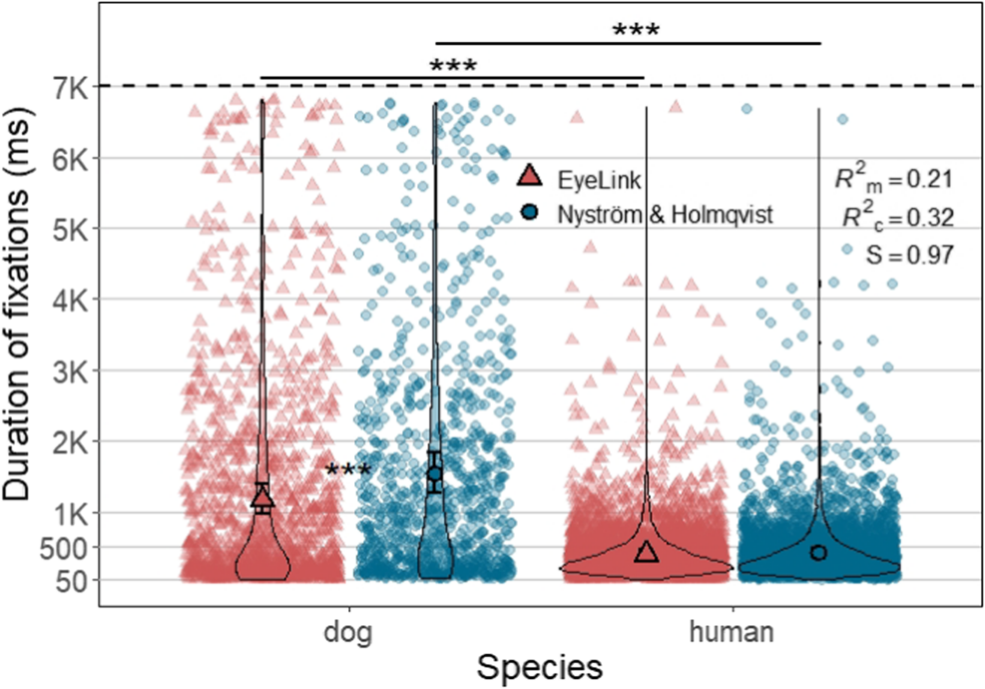
Each violin plot, demonstrating kernel probability density, depicts the distribution of fixation duration for human or dog data classified by either algorithm. Note the difference in the widths of the dog plots between two algorithms especially in the range below 500 ms. *Solid shapes* (a triangle or a circle) and *error bars* overlaid on the violin plots depict marginal means and 95% confidence intervals (± 1.96 ∗*SE*), respectively. Individual data points are indicated by triangles and circles in softened color. Note that fixations shorter than 50 ms were not included in the analysis

As can be seen in Fig. 11, statistical tests showed that species, algorithm and also their interaction significantly affected duration of fixations (interaction: X^2^(1) = 25.65, *p* < .0001, *S* = 21.22). Fixations classified by the Nyström and Holmqvist (2010) algorithm were significantly longer than fixations classified by the EyeLink algorithm (X^2^(1) = 41.79, *p* < .0001, *S* = 33.2). However, pairwise comparisons results showed that this difference between algorithms was significant only for dog fixations (Z = 6.47, *p* < .0001, *S* = 31.61) with effect size *d* = 0.28, 95% CI [0.20, 0.37]. The average duration of a dog fixation classified by the Nyström and Holmqvist (2010) algorithm was 1529 ms (*SE* = 143 ms), while that of the EyeLink algorithm was 1159 ms (*SE* = 107 ms). For human fixations, those were 413 ms (*SE* = 36 ms) for the Nyström and Holmqvist (2010) algorithm, and 398 ms (*SE* = 34 ms) for the EyeLink algorithm.

**Fig. 12:**
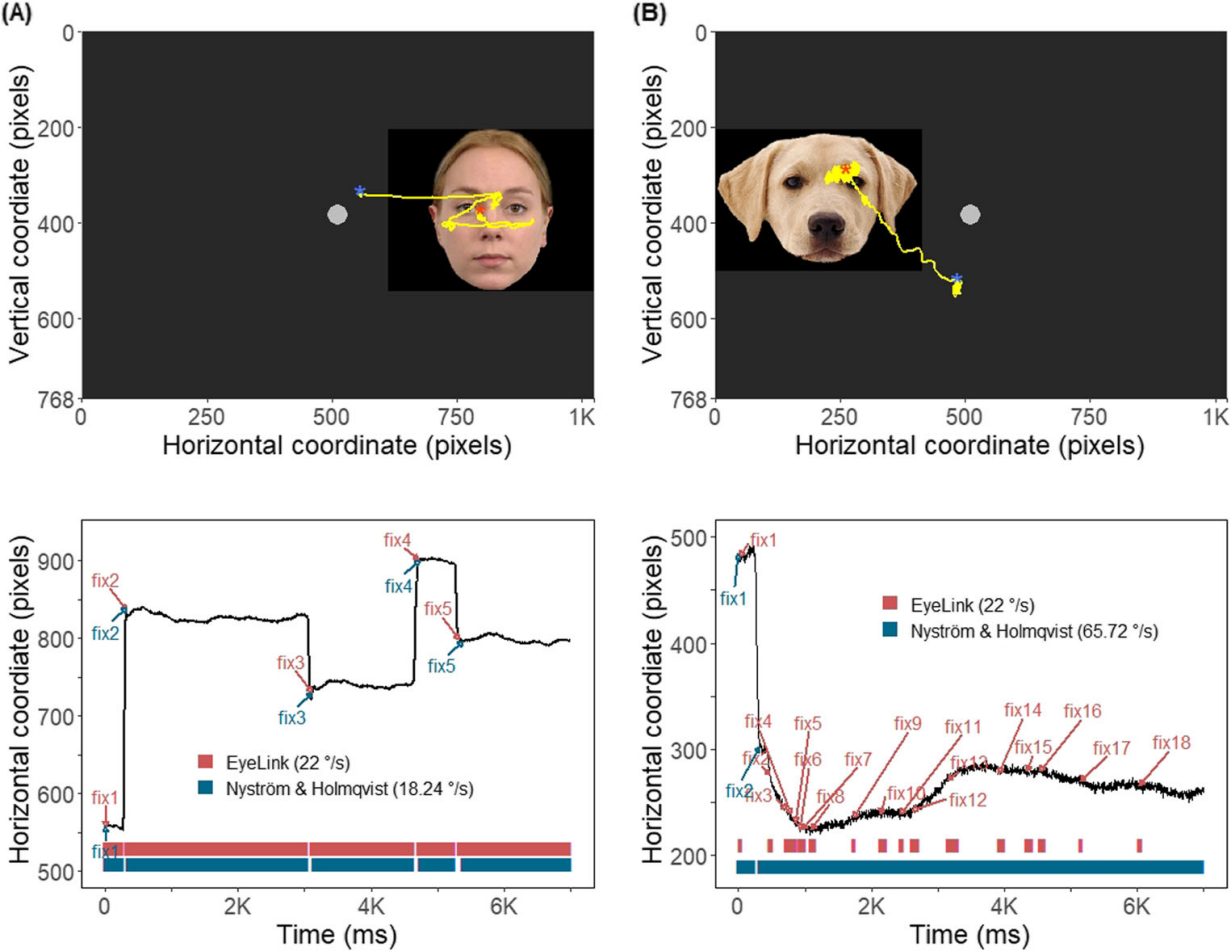
Panels **A** and **B** demonstrate an example of how similarly (panel **A**) or differently (panel **B**) the EyeLink and Nyström and Holmqvist (2010) algorithms classified eye-movement events in the same data depending on the quality of the data being classified. The two panels, composed of a scanpath plot (*top half* ) and a horizontal gaze coordinate plot (*bottom half* ), show data of a trial for a different dog. As shown in the lower plot of panel **A**, when classifying data of good quality, the classified fixation results of both algorithms were almost identical. On the other hand, when the data quality is lower (panel **B**), the results differed greatly: the EyeLink algorithm classified many more and much shorter fixations (eight fixations in the range of 50 to 160 ms and ten artefactual fixations shorter than 50 ms) than the two fixations (240 and 6672 ms) classified by the Nyström and Holmqvist (2010) algorithm. The velocity thresholds (^∘^/s) Nyström and Holmqvist (2010) algorithm used for each trial and the fixed EyeLink algorithm threshold (22 ^∘^/s) are indicated in the legend. A *blue* and a *red star* in each scanpath plot mark the start and end data point of each scanpath, respectively. The horizontal coordinate plots are labeled with the onset of the fixations identified by each algorithm, which correspond to the bar segments at the bottom

Further, we have compared the duration distribution of the fixations between the two algorithms. The results of two-sample Kolmogorov–Smirnov and Wilcoxon tests were significant only for dog fixation duration data revealing that the dog fixations classified by the two algorithms do not belong to the same distribution function (Kolmogorov-Smirnov: D = 0.14, *p* < .0001, *S* = 29.73; Wilcoxon: W = 493834, *p* < .0001, *S* = 37.43). Specifically, as demonstrated by the violin plots in Fig. 11, the data produced by the EyeLink algorithm had a relatively larger proportion of dog fixations in shorter duration range (< 500 ms) than the Nyström and Holmqvist (2010) algorithm. Panel B in Fig. 12 shows an example of such a case, where the EyeLink algorithm produced more and shorter fixations than the Nyström and Holmqvist (2010) algorithm for the same input data.

#### Fixation stability

**Fig. 13:**
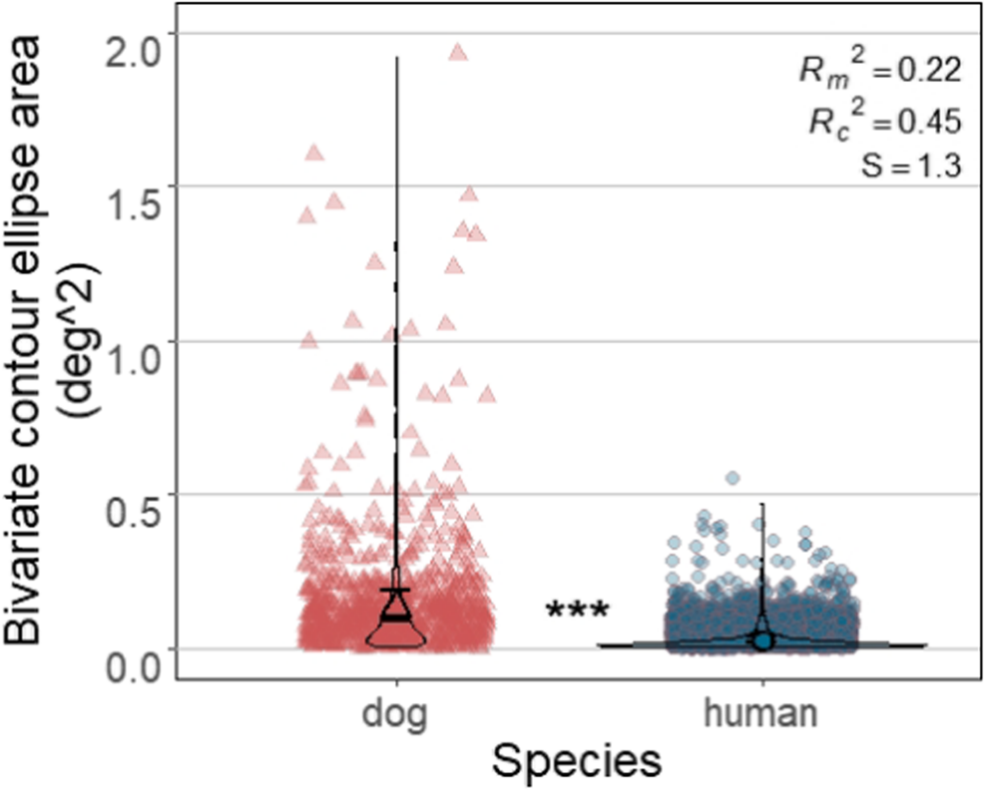
Each violin plot, demonstrating kernel probability density, depicts the distribution of BCEA values calculated for human and dog fixations classified by the Nyström and Holmqvist (2010) algorithm. *Solid symbols* and *error bars* overlaid on the violin plots depict marginal means and 95% confidence intervals (± 1.96 ∗*SE*), respectively. *Background symbols* indicate data used in the model. For visualization purpose, one dog fixation with BCEA value over 5 pixels^2^ is not shown in the plot

The bivariate contour ellipse area (BCEA) values of dog fixations were on average significantly larger than those of human fixations (Z = 6.84, *p* < .0001, *S* = 36.84; dogs: *M* = 0.13 *deg*^2^, *SE* = 0.02 *deg*^2^; humans: *M* = 0.02 *deg*^2^, *SE* = 0.004 *deg*^2^) indicating that the eye-movement data samples that comprise a dog fixation are significantly more dispersed, i.e., less stable than those that comprise a human fixation (Fig. 13). There was also more variation in BCEA values of dog fixations than human fixations (dogs: *SD* = 0.29 *deg*^2^, humans: *SD* = 0.03 *deg*^2^).

#### Velocity thresholds

**Fig. 14:**
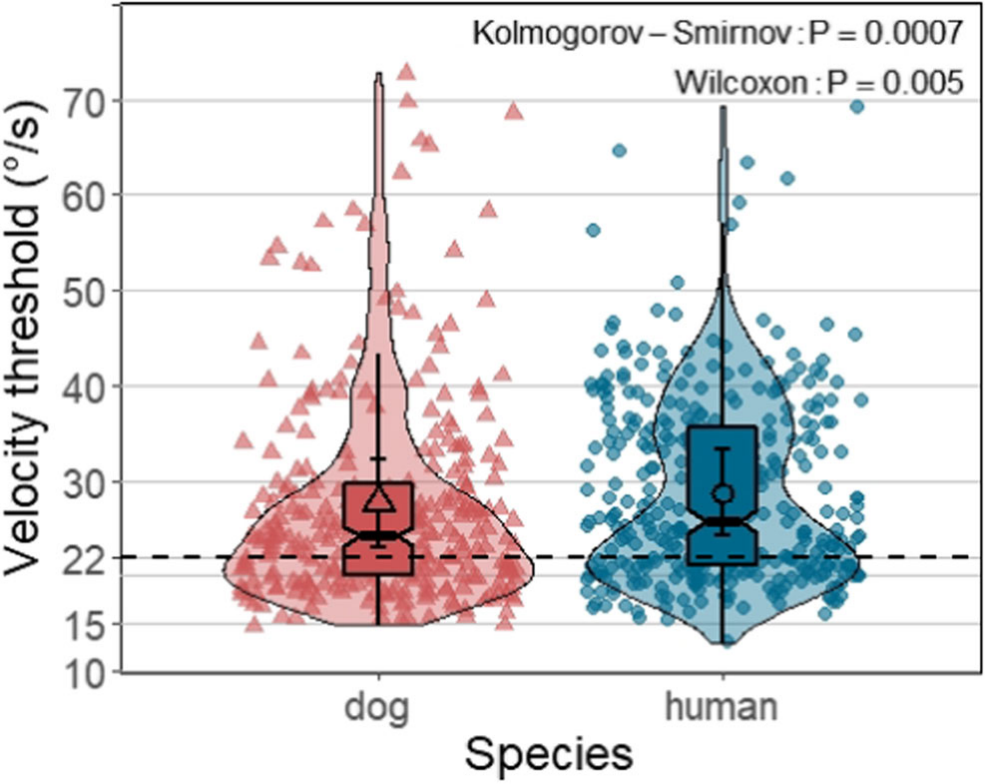
Each violin plot, demonstrating kernel probability density, depicts the distribution of velocity thresholds the Nyström and Holmqvist (2010) algorithm used to classify eye-movement events of dog and human data per trial. The lower and upper hinges of the notched box plots depict 25% to 75% inter-quantile ranges (IQRs) of dog and human data, respectively, with the ranges of their whiskers representing observation greater than or equal to the hinges ± 1.5 * IQR. Each notch of a box plot displays a confidence interval around the median which is based on the median ± 1.58*IQR/*n*. *Solid shapes* (a triangle or a circle) and *error bars* overlaid on the violin plots depict marginal means and 95% confidence intervals (± 1.96 ∗*SE*), respectively. *Shapes in softened color* in the background indicate threshold used for classifying the data of each trial. Note the wide range of variety in the thresholds the Nyström and Holmqvist (2010) algorithm used for both species data. The *black dashed line* indicates the velocity threshold of the EyeLink algorithm: 22^∘^/s

The mean velocity thresholds the Nyström and Holmqvist (2010) algorithm used for the two species were very similar (for human data: 28.7 ^∘^/s, *SE* = 2.16 ^∘^/s; for dog data: 27.7 ^∘^/s, *SE* = 2.24 ^∘^/s), yet the thresholds used by the Nyström and Holmqvist (2010) algorithm showed a slightly greater range for dog data (dog data: from 14.78 to 72.78 ^∘^/s; human data: from 13.05 to 69.3 ^∘^/s), and more trials of dogs (22 trials) had velocity thresholds over 50 ^∘^/s than humans (eight trials) as shown in Fig. 14. Therefore, two-sample Kolmogorov–Smirnov and Wilcoxon tests revealed that the thresholds used for dog and human data do not belong to the same distribution function (Kolmogorov–Smirnov: D = 0.16, *p* = .0003, *S* = 11.81); (Wilcoxon: W = 60015, *p* = .004, *S* = 7.97).

#### Three common fixation-related dependent variables

**Fig. 15:**
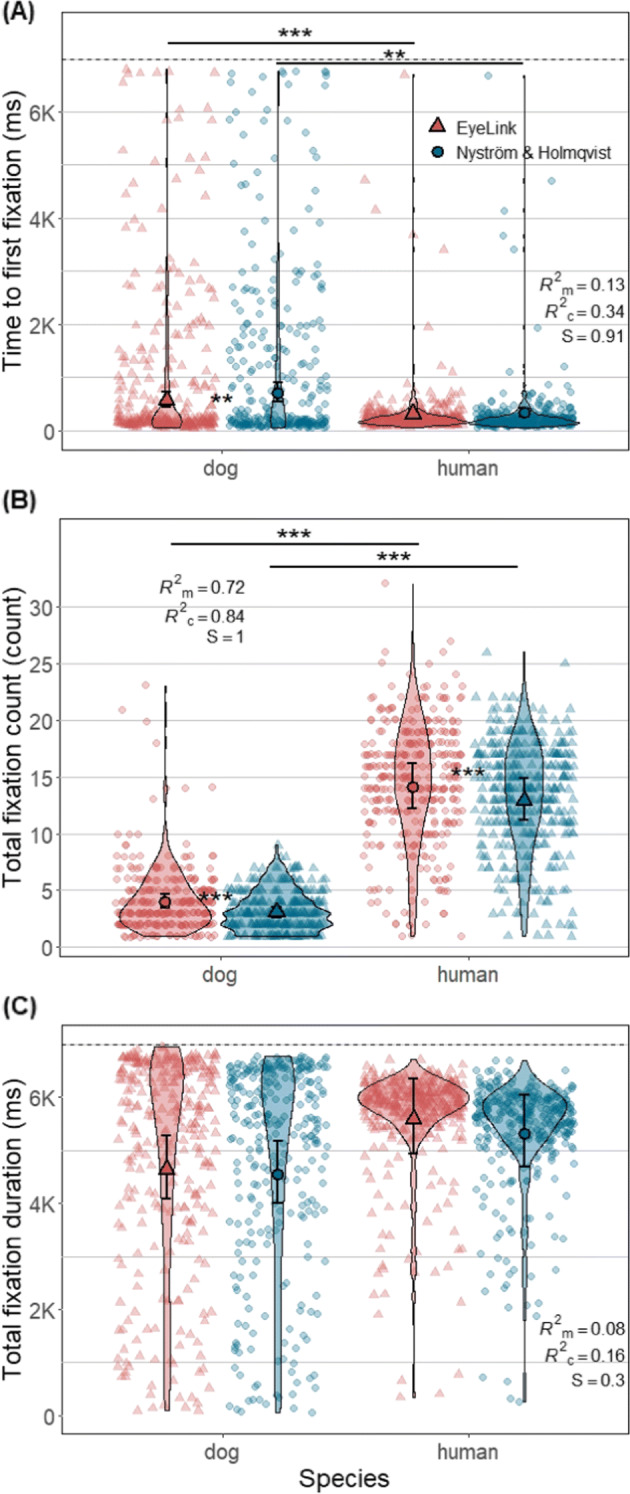
Each violin plot, demonstrating kernel probability density, depicts the distribution pattern of one of the three fixation-related measures per trial (plot A: Time to first fixation; plot B: Total fixation count; plot C: Total fixation duration) derived from dog or human fixations classified by either algorithm. The *black dashed line* in panels **A** and **C** indicates stimulus presentation duration. *Solid symbols* (a triangle or a circle) and *error bars* depict marginal means and 95% confidence intervals (± 1.96 ∗*SE*), respectively. *Smaller symbols* of the same shape in softened color in the background indicate trial data used for the analysis. Note that fixations shorter than 50 ms were not included in the analysis

For both algorithms, the time to first fixation of dogs was significantly larger than that of humans (EyeLink: Z = 3.29, *p* = 0.003, *S* = 8.38; Nyström and Holmqvist (2010): Z = 4.18, *p* = 0.0001, *S* = 13.29) (Fig. 15A). Overall, time to first fixations classified by Nyström and Holmqvist (2010) was larger, yet the difference between algorithms was significant only for dog fixations (Z = 3.09, *p* = 0.007, *S* = 7.16). The estimated mean latency of dog first fixations classified by the Nyström and Holmqvist (2010) algorithm was 705 ms (*SE* = 90.6 ms), while that of the EyeLink algorithm was 560 ms (*SE* = 71.6 ms). For human fixations, those were 336 ms (*SE* = 41.1 ms) for the Nyström and Holmqvist (2010) algorithm, and 313 ms (*SE* = 38.3 ms) for the EyeLink algorithm.

For both algorithms, the total fixation count of dogs was significantly smaller than that of humans (EyeLink: Z = 11.75, *p* < .0001, *S* = 87.59; Nyström and Holmqvist (2010): Z = 13.32, *p* < .0001, *S* = 117.12) (Fig. 15B). Overall, the fixation count based on the EyeLink algorithm fixations was significantly higher for both species, yet comparing the effect sizes, the algorithm difference of only dog fixations had practical significance (dogs: Z = 6.37, *p* < .0001, *S* = 30.78, *d* = 0.28, 95% CI [0.19, 0.36]; humans: Z = 4.40, *p* < .0001, *S* = 14.9, *d* = 0.09, 95% CI [0.05, 0.13]). The estimated mean fixation count of dogs classified by the EyeLink algorithm was 4.01 (*SE* = 0.32), while that of the Nyström and Holmqvist (2010) algorithm was 3.04 (*SE* = 0.25). For human fixations, those were 14.08 (*SE* = 1.02) for the EyeLink algorithm, and 12.89 (*SE* = 0.93) for the Nyström and Holmqvist (2010) algorithm.

The total fixation duration of dogs was not significantly lower than that of humans for both algorithms (Fig. 15C). Also, the difference between algorithms was not significant for both species. The estimated mean total fixation duration of dogs classified by the EyeLink algorithm was 4642 ms (*SE* = 306 ms), while that of the Nyström and Holmqvist (2010) algorithm was 4548 ms (*SE* = 300 ms). For humans, those were 5588 ms (*SE* = 359 ms) for the EyeLink algorithm, and 5320 ms (*SE* = 342 ms) for the Nyström and Holmqvist (2010) algorithm. We have further compared the distribution functions of the total fixation duration data of each algorithm between humans and dogs, and also the data of each species between the two algorithms. Two-sample Kolmogorov-Smirnov tests revealed that the data of dogs and humans do not belong to the same distribution function regardless of the algorithms used for producing the data (EyeLink: D = 0.29, *p* < .0001, *S* = 38.54; Nyström and Holmqvist (2010): D = 0.26, *p* < .0001, *S* = 30.9). Furthermore, within each species, the total fixation duration data of the EyeLink and Nyström and Holmqvist (2010) algorithms also did not belong to the same distribution function (dogs: D = 0.12, *p* = 0.04, *S* = 4.64; humans: D = 0.25, *p* < .0001, *S* = 30.63). As Fig. 15C shows, while most data of humans were aggregated between 5000 ms and 7000 ms, such aggregation was not seen in dog data which is relatively fairly distributed from 0 to 7000 ms. Similarly, dog data had many more data points near zero and below 2000 ms than human data.

## Discussion

Our results show that several morphological characteristics of the face of dogs interfere with the image processing and gaze estimation operations of P–CR eye trackers, thereby increasing the chances of data quality reduction in dogs, as compared to humans. Furthermore, we found that dogs blinked less often than humans but their blink was longer. Whether for morphological reasons, due to more head movement, or difficulties in calibration, our results support the indications of lower quality in dog eye-tracking data reported by previous studies and confirm that the quality of dog eye-tracking data is overall lower than that of humans. Classifying fixations with dog data was more sensitive to algorithm choice. That is, there were significant between-algorithm differences for dog data, but not for human data in the two fixation classification outputs, proportion of artefactual fixations and average duration of a fixation. Furthermore, most of our fixation-related measures were affected by the choice of algorithm: time to first fixation and total fixation count differed between our two algorithms in dogs but not in humans. Two measures derived from the Nyström and Holmqvist (2010) algorithm that reflect noise in data further supported lower quality of dog data compared to human data. Dog fixations were less stable than human fixations, and the Nyström and Holmqvist (2010) algorithm needed to use many more extreme velocity thresholds with dog data than with human data. In the following, we discuss our findings in detail.

### Subject selection and data collection

Using the eye-video of a P–CR eye-tracking system, we have observed that several morphological characteristics of dogs interfere with the performance of the eye tracker, mostly by blocking the view of the pupil or corneal reflection or by interfering with reliable determination of their centers. Our results demonstrate the importance of recognizing and familiarizing oneself with these morphological characteristics and their impact before conducting an eye-tracking experiment with dogs to be able to avoid their detrimental effects on data quality as much as possible. We recommend experimenters to thoroughly examine each dog for the characteristics when they recruit dogs for eye-tracking experiments. Note that while we used the eye-video images to examine and show how the characteristics could cause tracking interruptions, a thorough naked eye examination should in practice be sufficient for spotting problematic morphology in most cases.

In practice, it would be best to recruit dogs with shorter and more downward-pointing snout, less droopy eyelids, bigger eye clefts, less-spiky or shorter facial hair, lighter-colored eyelid margins, and minimally visible third eyelid. Additional preparations may also be necessary for successful eye tracking, or improving the quality of the recorded data. For example, similar to eye make-up removal that is commonly practiced in human eye tracking, researchers could carefully flush out excess mucus on the eye surface of dogs and clean the facial area surrounding the eyes using sterile buffered saline and gauze, possibly with support of a veterinarian. Further, some of the spiky and long hairs on the snout could be cautiously cut away if they interfere with the eye tracker’s view of the dog’s eyes. It is plausible that tracking both eyes of a dog subject at the same time is difficult when the dog subject is brachycephalic (short-nosed), even without obvious physical characteristics that could be expected to cause track loss, because of the generally more laterally oriented eyes in such dogs. An inability to conduct binocular recordings would limit investigations of binocular eye movements such as vergence. Further, we note that adjusting the luminance of the stimulus might be beneficial to eye-tracking data quality. Considering that the pupils of dogs are relatively large compared to the size of their eye cleft, using bright stimuli and recording in a relatively well-lit environment would make the pupil smaller and, thus reduce the chance that the pupil is (partially) covered by surrounding ophthalmic structures (Drewes et al., 2014; Holmqvist, 2015). This has been shown also to reduce variability in eye-movement data of human adults (Hooge et al., 2019). However, note that it is important to keep the luminance of the screen and the illumination of the environment the same during the calibration and validation procedures and the stimulus viewing task to prevent pupil size-related offsets in the estimated gaze location (Wyatt, 2010; Drewes et al., 2012; Choe et al., 2016; Hooge et al., 2021).

We have also shown for the first time that dogs blink differently than humans. Dogs were unlikely to blink during our seven-second long recording, while humans likely blinked at least once. The average duration of dog blinks was however longer than that of humans. The results suggest that dog data may be less likely affected by blink incidences than human data as long as stimulus presentation remains as short as maximum several seconds. At the same time we found bigger variation in blink duration among dogs than humans. This may in turn be one factor affecting the greater variability in the amount of data loss and the number classified eye movements for dogs compared to humans. On the other hand, as we have discussed in the subsection Other conditions in the Result section, dogs are known to make partial blinks that can be falsely classified as saccades by current algorithms. Filtering out partial blinks from the data may be possible by detecting rapid changes in the pupil size signal provided by most P-CR eye trackers.

Additionally, unlike humans, dogs do not have sweat glands on most parts of their skin. Dogs thus mainly pant when they need to reduce their body temperature. This makes recording eye-movement data from dogs at relatively high environmental temperatures problematic, as the head of the dog would constantly slightly move up and down, inducing potential inaccuracies in the eye-movement data. We thus advise to avoid recording during warm weather conditions, or take measures to maintain cooler body temperature during the experiment, since this would help reduce their panting and thus this head movement.

Many dog eye-tracking studies, including ours, have used a chinrest. The effect of a chinrest on data quality has not been systematically investigated with dogs. In our experience using a chinrest with dog subjects was useful, because it helped to minimize lateral translations and rotations of the dog’s head, which are known to negatively affect data quality in human participants (e.g., Hessels et al., (2015) and Niehorster et al., (2018)). To the best of our knowledge so far only one study has explored the effect of using a chinrest on the quality of data recorded from highly experienced human participants who were instructed to sit still. While this study reported mixed results (Holmqvist et al., 2021), we expect that for dogs having a chinrest is likely more important than for humans since it is significantly more difficult to instruct dogs to minimize their head movement.

While our study used a stationary eye-tracking system, head-mounted eye-tracking systems which have successfully been used with cats (Einhauser et al., 2009) and non-human primates (Mennie et al., 2014) might be a useful alternative to stationary systems for doing eye tracking with dogs. Already some systems have a supplementary head tracking option, yet the head-mounted systems would be especially useful if researchers want to record eye movement of dogs that engage in exploratory behavior outside of the experimental room. There has been one experimental attempt to develop such devices which used an existing infrared eye-tracking system and placed it on a muzzle with minor modification (Williams et al., 2011), although we are not aware of further published studies that report data collected using the system. Head tracking-only systems were also developed for tracking a dog’s field of view (Rossi et al., 2014), however we see such techniques as more suitable for studying the gaze behavior of animal species such as owls that make only head movement for shifting gaze in contrast to dogs and humans (Ohayon et al., 2008).

### Analysis of dog eye-tracking data

#### Understanding the impact of data quality on eye-tracking data analysis

Our results show that dog data are more susceptible to algorithm choice because of its relatively lower quality compared to human data. First, only for dog data the EyeLink classification algorithm produced a significantly higher proportion of artefactual (< 50 ms) fixations than the Nyström and Holmqvist (2010) algorithm, while for human data this outcome did not depend on the algorithms. Excluding these artefactual fixations, we then examined the duration of the classified fixations. Similar to the result of proportion of artefactual fixations, only for dog data a difference between algorithms occurred. The results of fixation stability and velocity threshold, the two measures derived from Nyström and Holmqvist (2010) algorithm, further support that the quality of dog data is lower than human data. First, dog fixations classified by Nyström and Holmqvist (2010) algorithm were more dispersed than human fixations. This finding that dog data is noisier is likely at least partially because dogs make more frequent head movement than humans or due to their unique eye morphology. On the other hand, the higher BCEA values may indicate that dog fixations have more fixational drift than human fixations, but we cannot rule out that it is due to dogs making more head movements during the task despite being positioned on a chinrest. Further investigation of fixational eye movement in dogs needs to clarify to what extent higher dispersion in dog fixations reflects true species-specific differences in eye-movements of dogs and humans.

Second, the results of velocity thresholds that the Nyström and Holmqvist (2010) algorithm used for classifying dog and human data showed that, while the average thresholds for dog and human data did not differ significantly, there were many more extreme thresholds (over 50 ^∘^/s) for dog than human data. As the Nyström and Holmqvist (2010) algorithm adaptively uses higher velocity thresholds for noisier trial data, the finding indicates that dog data had more trials that were noisier than human data.

Wass et al., (2014) have shown how differences in data quality influence the results of key dependent variables in eye-tracking studies. One of the dependent variables they examined was the time to first fixation, and they suggested that more data loss, which often occurred in their infant subjects, may manifest as longer latency. Our results of time to first fixation supports this. Visually examining the trial data in Fig. 15A, it can be noticed that the latency of the first fixations is largely concentrated on the lower range (< 500 ms) for both species, yet dogs have more first fixations dispersed in higher latency range than humans. This seems to indicate that the larger estimated mean time to first fixation of dogs is biased by the data in higher latency range rather than that dogs overall make first fixations more slowly than humans. It is unclear if the data in higher latency range is due to lapses of attention or data quality in dogs. However, it should be noted that our latency measure has the limitation that dogs, in contrast to humans, could not be clearly instructed to start each trial with fixating on the center of the screen.

Certain fixation-related measures are inevitably influenced by the average duration of the fixations (Orquin and Holmqvist, 2017). One of them is total fixation count, which one would expect to be lower for trials of constant duration if the average fixation duration is higher. We indeed saw this in our data, where the total fixation count of dogs was significantly lower than that of humans who have significantly shorter fixations than dogs. Also, the pattern of statistical results for total fixation count was identical to that for fixation duration i.e., while the counts of both algorithms for dogs were significantly lower than those for humans, only dogs’ total fixation counts were significantly different between algorithms. The two fixation-related dependent variables above are popularly used in eye-tracking studies as key dependent variables in combination with areas of interest (AOIs) on the stimuli planned by researchers, as the results of the variables are used to gauge subjects’ attentional status on the AOIs. Our results show how important it is to put more effort to collect better quality data and to use data processing tools more robust to lower quality data in order to increase the reliability of the results and, consequently, the conclusions of dog eye-tracking studies.

Nevertheless, not all eye-tracking data of dogs are of lower quality. It should be noted that, while the quality of dog data is overall poorer than that of humans, there is also much more variation in the quality across individuals and their trials in dogs than humans. We have shown an example of such with scan path visualizations of two different dogs in Fig. 12, where panel (A) visualizes the data of a dog with the data quality that is roughly equal to that of most human subjects, while panel (B) visualizes a very different case. The classification results of the dog data in panel (A) did not differ much between algorithms as shown with a bar segment plot below the scan path plot. This particular dog not only passed the subject selection criteria by not having major morphological features that could interfere with tracking, but also was one of the highest performing dogs in chinrest and calibration training. This emphasizes again the value of selecting subjects based on their morphological characteristics and training performance.

#### Understanding the impact of algorithms on eye-movement event classification of dog data

The results discussed above highlight the impact of algorithm choice on eye-movement event classification in dog data. Following our results of artefactual fixation ratio, only EyeLink algorithm produced significantly different ratios between species. This difference in how the EyeLink and the Nyström and Holmqvist (2010) algorithms performed can possibly be due to the fixed velocity threshold employed by the EyeLink algorithm, while the adaptive thresholding procedure used by the Nyström and Holmqvist (2010) algorithm enabled it to adapt to differences in data quality between and within the data sets of the two species, and also the differences in eye-movement characteristics between the two species or individuals.

Similar differences in the results derived from the differences among algorithms can also be observed when we compare our results to that of other studies. The average duration of a dog fixation calculated based on the fixations classified by the Nyström and Holmqvist (2010) algorithm in this and our previous study (that used a larger data set) differ greatly from those other previous studies reported. The average duration of a fixation reported by our previous study was 1593 ms (Park et al., 2020), which is longer than the 827 ms reported by Barber et al., (2016) and much longer than the 214 ms reported by Somppi et al., (2012). The combination of the varying quality of dog data and the different algorithms used across the studies are likely the cause of this difference. Therefore, we re-emphasize the point made by others (e.g., Holmqvist et al.,, 2011; Oakes, 2010) that to make results comparable across dog and human eye-tracking studies, it is crucial that future dog eye-tracking studies describe in detail their eye-movement event classification procedure, including the algorithm they used, any parameter settings employed, and the procedure for filtering out artefactual eye movements, if any. Holmqvist et al., (2022) present advice on how to report these method details.

The longer average duration of dog fixations needs to be taken into account when designing dog eye-tracking experiments. So far, many dog-eye-tracking studies have used stimulus presentation durations as short as 1500 ms (Table 1). We argue that if researchers aim to observe more than three fixations, a minimum duration of 5000 ms is needed for stimulus presentation.

Our findings also highlight the importance of post hoc filtering of artefactual eye-movement events, such as too short fixations and saccades. Wass et al., (2013) compared how two algorithms classified eye-movement events in infant data, and reported that their custom-made algorithm that had post hoc filtering of artefactual eye-movement events performed better than an algorithm provided by the eye-tracker’s manufacturer without post hoc filtering. Accordingly, SR Research, the manufacturer of the EyeLink, recommends post hoc filtering of artefactual eye movements in addition to trying out different sets of thresholds in order to find optimal threshold values for a given data set.

Five different eye-tracking systems from four manufacturers have been used across the dog-tracking literature (see Table 1), and it should be asked if our results are limited to work done with an EyeLink eye-tracker, or also have bearing on studies performed with other systems. While it is not possible to predict what the data quality of each system would be when tracking dog eyes without a direct comparison study, a statement can be made regarding the event classification algorithms that these other manufacturers provide. Specifically, the default algorithms of Tobii Pro Lab and SMI BeGaze are to the best of our knowledge also velocity-based algorithms that work with a fixed velocity threshold. As such, assuming similarly lower data quality in dogs than in human eye-tracking data with these systems, we would expect a similar pattern of results as presented here for the EyeLink algorithm.

While based on our results, we recommend using a noise-adaptive algorithm, in extreme cases where such automatic approaches are not available, manual coding of eye-movement events might be considered as an alternative method for event classification of dog data (but see Hooge et al.,, 2018). While we echo the advice of Hooge et al., (2018) that manual classification of eye-movement data should only be performed if no other options are available, it is worth noting that manual classification is relatively tractable in case of dog eye-movement data, because the number of subjects and the total duration of experiments are usually limited due to the short attention span of dogs.

## Data Availability

The custom Nyström and Holmqvist (2010) algorithm as implemented by Niehorster et al., (2015) is available at https://github.com/dcnieho/NystromHolmqvist2010.
